# The next generation of alginate dressings: recent innovations for chronic wound healing

**DOI:** 10.1093/rb/rbag099

**Published:** 2026-05-21

**Authors:** Eduarda Pinto, Cristina C Barrias, Judite Novais Barbosa

**Affiliations:** I3S—Instituto de Investigação e Inovação em Saúde, Universidade do Porto, Porto 4200-125, Portugal; INEB—Instituto de Engenharia Biomédica, Porto 4200-125, Portugal; ICBAS—Instituto de Ciências Biomédicas Abel Salazar, Universidade do Porto, Porto 4050-313, Portugal; I3S—Instituto de Investigação e Inovação em Saúde, Universidade do Porto, Porto 4200-125, Portugal; INEB—Instituto de Engenharia Biomédica, Porto 4200-125, Portugal; ICBAS—Instituto de Ciências Biomédicas Abel Salazar, Universidade do Porto, Porto 4050-313, Portugal; I3S—Instituto de Investigação e Inovação em Saúde, Universidade do Porto, Porto 4200-125, Portugal; INEB—Instituto de Engenharia Biomédica, Porto 4200-125, Portugal; ICBAS—Instituto de Ciências Biomédicas Abel Salazar, Universidade do Porto, Porto 4050-313, Portugal

**Keywords:** alginate, chronic wounds, bioengineering, regeneration, wound management

## Abstract

Chronic wounds remain a major clinical challenge with a huge associated socioeconomic burden. Therefore, multifunctional biomaterials that can simultaneously manage exudate, modulate inflammation, prevent infection and promote tissue regeneration are extremely needed. Alginate, a naturally derived and highly biocompatible polysaccharide, has long been used in the clinic for advanced wound care due to its intrinsic gel-forming ability and capacity to maintain a moist healing environment. However, commercially available products are primarily designed to absorb exudate and physically protect the wound, while offering limited support for other key therapeutic goals, including modulation of inflammation and stimulation of tissue repair and regeneration. This review highlights the most recent innovations aimed at enhancing alginate’s therapeutic potential, either through physicochemical modifications of hydrogels or by expanding beyond traditional hydrogel formats into novel alginate architectures that offer improved tissue integration, tunability and controlled release of substances. The review also outlines synergistic formulations combining alginate with other biomaterials, bioactive molecules and cell-derived products to enhance its mechanical properties and antimicrobial performance, as well as to address immunomodulation and promote regeneration in chronic wounds. Together, these emerging approaches underscore alginate’s versatility and potential as a multifunctional platform for next-generation chronic wound therapies.

## Graphical Abstract

**Graphical Abstract:**
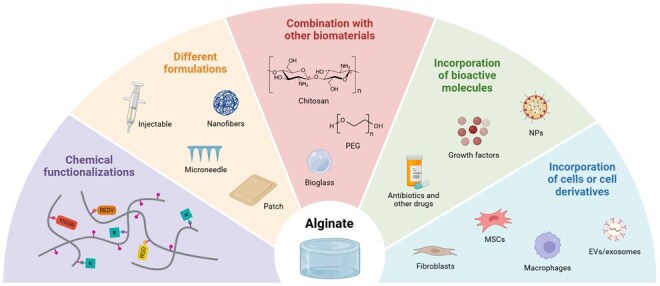

## Alginate

Alginate is a naturally occurring biomaterial commonly used as a hydrogel for several biomedical applications, including wound healing. It can be extracted from brown algae and bacteria, with algae being the main source of commercially available alginates and, consequently, of most clinical-grade formulations [1]. Still, it is essential to ensure that the manufacturing and purification processes are appropriate for the intended application, as different purification levels are available and some preparations may contain residual contaminants and endotoxins [2–4]. Nonetheless, its natural origin, biodegradability and potential for sustainable sourcing and recycling characterize alginate as an environmentally friendly polymer [5].

**Figure 1:**
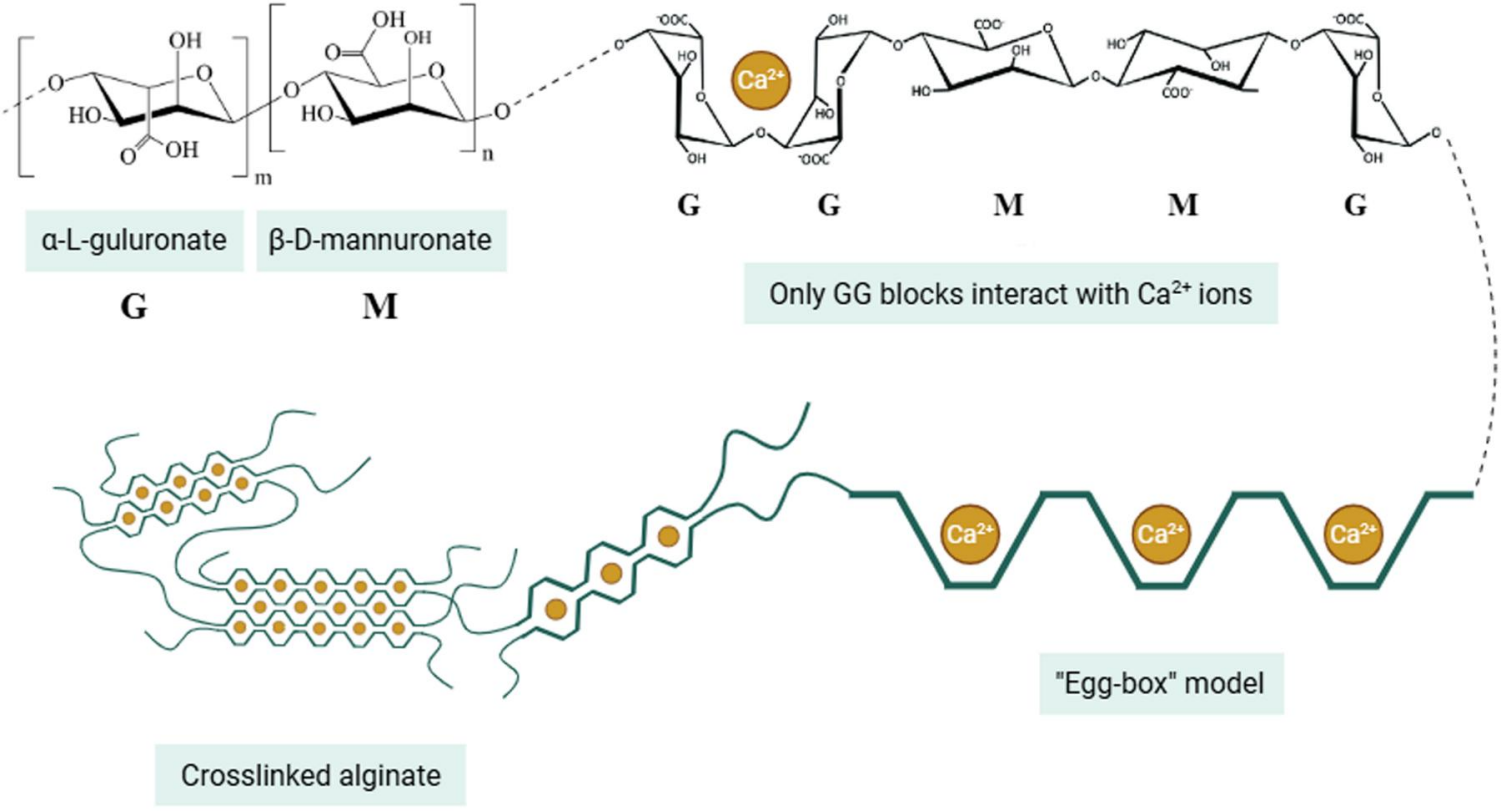
Schematic representation of alginate monomers, namely α-L-guluronate (G) and β-D-mannuronate (M), and ionically crosslinked alginate (e.g. with calcium ions). Adapted from [6] and [7], created with ‘BioRender.com’.

This hydrophilic anionic polysaccharide is composed of (1,4)-linked α-L-guluronate (G) and β-D-mannuronate (M) residues arranged in homopolymeric (GG and MM) and heteropolymeric (MG/GM) blocks (Figure 1). The ratio and sequential distribution of G and M units vary depending on the alginate source and determine its chemical, physical and biological properties [8, 9]. In addition, the high density of hydroxyl (–OH) and carboxyl (–COOH) groups confers marked hydrophilicity and water absorption capacity, which are essential for maintaining a moist wound environment [10]. For biomedical applications, alginate hydrogels are typically prepared as 0.5–3% (w/v) alginate solutions through ionic gelation with divalent cations (Ca^2+^, Ba^2+^ or Mg^2+^) [11]. The most widely used approach is external gelation, in which alginate solutions or pre-formed dressings are exposed to calcium chloride (CaCl_2_) baths or sprays, leading to rapid crosslinking at the surface and the formation of mechanically robust hydrogels [12]. In contrast, internal gelation, commonly achieved using calcium carbonate (CaCO_3_) and glucono-δ-lactone (GDL), allows the gradual release of Ca^2+^ ions and the formation of more structurally uniform but generally softer hydrogels [13]. In both approaches, Ca^2+^ ions interact preferentially with G-blocks to create the so-called ‘egg-box’ structure, generating a crosslinked network (Figure 1). Although ionic crosslinking is the most prevalent method, gelation can also be achieved through several other strategies, including covalent crosslinking using glutaraldehyde or polyethylene glycol diacrylate (PEGDA), Schiff base reaction, photo crosslinking and thermal crosslinking [14].

### Biocompatible characteristics

Taking into account that alginate is abundantly available in nature and highly biocompatible, it has been widely used as a biomaterial in several fields, not only in medical applications but also in cosmetics, bioremediation and the food industry. Alginate-based products are generally recognized as safe (GRAS) and are approved by the Food and Drug Administration (FDA) for specific uses [8]. For wound healing applications, the main advantage of alginate is its ability to manage moderate-to-high exudate, together with its capacity to conform to irregularly shaped wounds [15]. It can also provide effective haemostasis in bleeding wounds by enhancing local clot formation and is generally considered non-immunogenic [1].

In addition, alginate hydrogels exhibit structural and biomechanical features that resemble certain aspects of the extracellular matrix (ECM), making them attractive scaffolds for cell embedding in a 3D environment. Furthermore, as will be discussed later in this review, alginate can be easily modified to tailor its mechanical, chemical and biological properties, thereby influencing cell behaviour and potentially improving wound closure [16]. Alginate hydrogels can also serve as drug delivery platforms, enabling controlled release profiles that can be adjusted according to specific applications [17]. For example, alginate hydrogels display pH-responsive behaviour: at higher pH values, such as those typically found in chronic wounds, deprotonation of carboxyl groups increases electrostatic repulsion within the polymer network, promoting swelling and the consequent release of loaded therapeutic agents [18, 19]. Conversely, at lower pH values, protonation reduces charge repulsion, leading to hydrogel shrinkage. Provided that these variations in ionization do not compromise the integrity of the polymer network, the swelling-shrinking process remains largely reversible, underscoring the versatility and functionality of alginate hydrogels [19].

## Chronic wounds

A wound typically undergoes a complex healing process comprising three main phases. First, an inflammatory phase begins with fibrin clot formation and proceeds to immune cell recruitment in response to the local release of cytokines, growth factors and damage-associated molecular patterns (DAMPs). Polymorphonuclear leukocytes (PMNs), macrophages and lymphocytes are initially responsible for the clearance of cellular debris and microorganisms, but later, PMNs exit the wound environment and macrophages switch to an M2, anti-inflammatory profile, which further induces re-epithelization by keratinocytes, stimulates fibroblast activity and promotes angiogenesis. At this point, a proliferative phase is initiated, and ECM assembly is essential to support cell migration, vascular network formation and ultimately wound closure. Finally, a remodelling phase occurs to enhance tissue integrity, mainly characterized by a conversion of previously secreted collagen type III fibres into organized type I fibres. A tightly regulated balance between matrix production and degradation is fundamental for the formation of a healthy scar and successful wound healing [20–22].

However, when a wound fails to progress through this sequence of phases and to close within an expected time frame, it is considered chronic and can ultimately lead to limb loss [23]. Chronic wounds usually remain trapped in the inflammatory phase and can be further classified as arterial, diabetic, pressure and venous ulcers according to their etiology [24]. Physiologically, chronic wounds are typically characterized by a persistent inflammatory state and an increased risk of infection, including the formation of antibiotic-resistant biofilms [25, 26]. Moreover, they are often associated with other underlying clinical conditions, such as diabetes, obesity, ageing and vascular insufficiency, that hinder repair progression and impair healing [20]. Due to their non-healing nature and constant need for care, chronic wounds negatively impact patients’ quality of life and represent a substantial socioeconomic burden [27].

### Commercially available alginate-based products for chronic wound treatment

The standard of care for chronic wounds primarily focuses on wound management rather than on the promotion of regeneration. Surgical debridement, meaning the surgical removal of non‐viable wound tissue, is frequently carried out, followed by antimicrobial treatment, when needed, and coverage of the wound with dressings that both protect the wound from external aggressions and absorb wound exudate [28, 29]. Taking into account their biocompatibility and high absorption capacity, alginate-based dressings have long been used in clinical wound care to manage heavy exudate [30], and examples of commercially available products can be found in Table 1. However, given the alginate bioinert nature, when used alone, it offers limited benefits for regeneration. In fact, evidence from systematic reviews and randomized controlled trials remains inconsistent, with several studies reporting no significant improvement in healing outcomes compared to alternative dressings, despite mentioning that there is a strong need for higher-quality clinical data [31–34]. This highlights the need for the development of advanced alginate-based products to improve clinical application and patient outcomes.

**Table 1:** Commercially available products for chronic wound management [1, 2, 30, 35, 36].

Name	Company	Composition
Kaltostat®	ConvaTec	Calcium and sodium alginate
Hyalogran®	ConvaTec	Sodium alginate and hyaluronic acid
Comfeel®	Coloplast	Calcium alginate and sodium carboxymethyl cellulose
Biatain®	Coloplast	Alginate and carboxymethyl cellulose
Purilon Gel®	Coloplast	Calcium alginate and carboxymethyl cellulose
SeaSorb®	Coloplast	Calcium alginate
Tegagen™	3M	Sodium alginate
Fibracol™ Plus	3M	Calcium alginate and collagen
NU-Derm™	Systagenix	Sodium alginate
Silvercel™	Systagenix	Alginate, carboxymethyl cellulose, nylon and silver
Algisite M®	Smith+Nephew	Calcium alginate
Melgisorb®	Mölnlycke	Calcium and sodium alginate
Pharma-Algi®	Pharmaplast	Calcium and sodium alginate
Algostéril®	Brothier	Calcium alginate
Urgosorb®	Urgo	Calcium and sodium alginate, carboxymethyl cellulose
Algicell® Ag	Integra	Calcium alginate and silver
Sorbsan®	Sorbsan	Calcium alginate
Algivon®	Advancis Medical	Calcium alginate and Manuka honey

Duciel et al. [2] evaluated *in vitro* the performance of six different commercially available alginate dressings and found that only Algostéril^®^ was non-cytotoxic, maintaining 91% fibroblast viability. The authors also assessed calcium (Ca^2+^) release from the dressings and concluded that it depended on both the initial percentage of calcium content (as some of the dressings also contained sodium alginate in their composition) and the M/G ratio. Since Ca^2+^ binds preferentially and strongly to G-rich blocks, alginate dressings with a higher proportion of GG sequences exhibited lower Ca^2+^ release, while MM-rich alginate showed higher Ca^2+^ release [2]. This analysis is particularly relevant considering that, when applied to wounds, alginate dressings exchange Ca^2+^ ions with Na^+^ ions present in exudate and blood. The released Ca^2+^ plays a critical role in the wound healing process by regulating several cell functions, including cell proliferation, migration and phagocytosis, as well as ECM remodelling and angiogenesis [37–39]. Concerning exudate management, although some of the tested dressings contained highly absorbent components (such as carboxymethylcellulose, sodium alginate or M-rich blocks) capable of retaining the liquid within their fibres, Algostéril exhibited the highest draining capacity, likely due to the presence of numerous lobes on the fibre surface [2].

The same group had previously studied the effect of some of these commercially available dressings on dermal fibroblasts, one of the key cell types involved in wound healing, and confirmed that the tested alginate-based dressings stimulated fibroblast migration and proliferation. Moreover, unlike the other dressings, Algostéril^®^ induced significant activation of transforming growth factor beta (TGF-β), leading to increased secretion of vascular endothelial growth factor (VEGF) and collagen [40]. They also tested the effect of Algosteril^®^ and Biatain^®^ on macrophage polarization and observed that both alginates promoted an M1 pro-inflammatory profile, with Algosteril^®^ additionally enhancing the secretion of chemokine ligand 18 (CCL18) [41]. In fact, Wu et al. [42] concluded that even varying M/G ratios of alginate alone are sufficient to influence macrophage polarization. More precisely, in Cu-loaded alginate, higher M/G ratios were shown to induce migration and an M1-like phenotype, whereas lower M/G promoted a shift toward an M2-like profile.

In an attempt to functionalize already existing commercial alginate dressings, Niculescu et al. [43] described a strategy to incorporate Zn^2+^ ions through direct ionic crosslinking on top of conventional alginate-coated dressings. Using this approach, they were able to maintain hydrogel integrity while increasing antimicrobial activity and significantly promoting wound closure and tissue regeneration in a rat burn model. These findings illustrate that even small modifications of alginate hydrogels can substantially improve their therapeutic performance in chronic wound management, with clear benefits for patients.

## The next generation of alginate-based dressings

In recent decades, the standard of care for chronic wounds has not changed significantly, remaining restricted to conventional treatment options that focus primarily on debridement and exudate management, with limited attention to active stimulation of tissue repair [44]. Alginate-based products, with their inherent versatility and biocompatibility, remain uniquely positioned to drive the next generation of wound therapies. Adding to exudate management and wound protection, these innovative alginate-based wound therapies should encompass three main features, namely the ability to (1) control inflammation, (2) promote tissue regeneration and (3) minimize the risk of infection. Accordingly, the following sections review some of the most recent advances involving alginate in combination with other biomaterials and bioactive molecules in an attempt to transform it from a passive material into a multifunctional active therapeutic platform for chronic wound treatment.

### Chemical functionalization of alginate for enhanced performance

**Figure 2:**
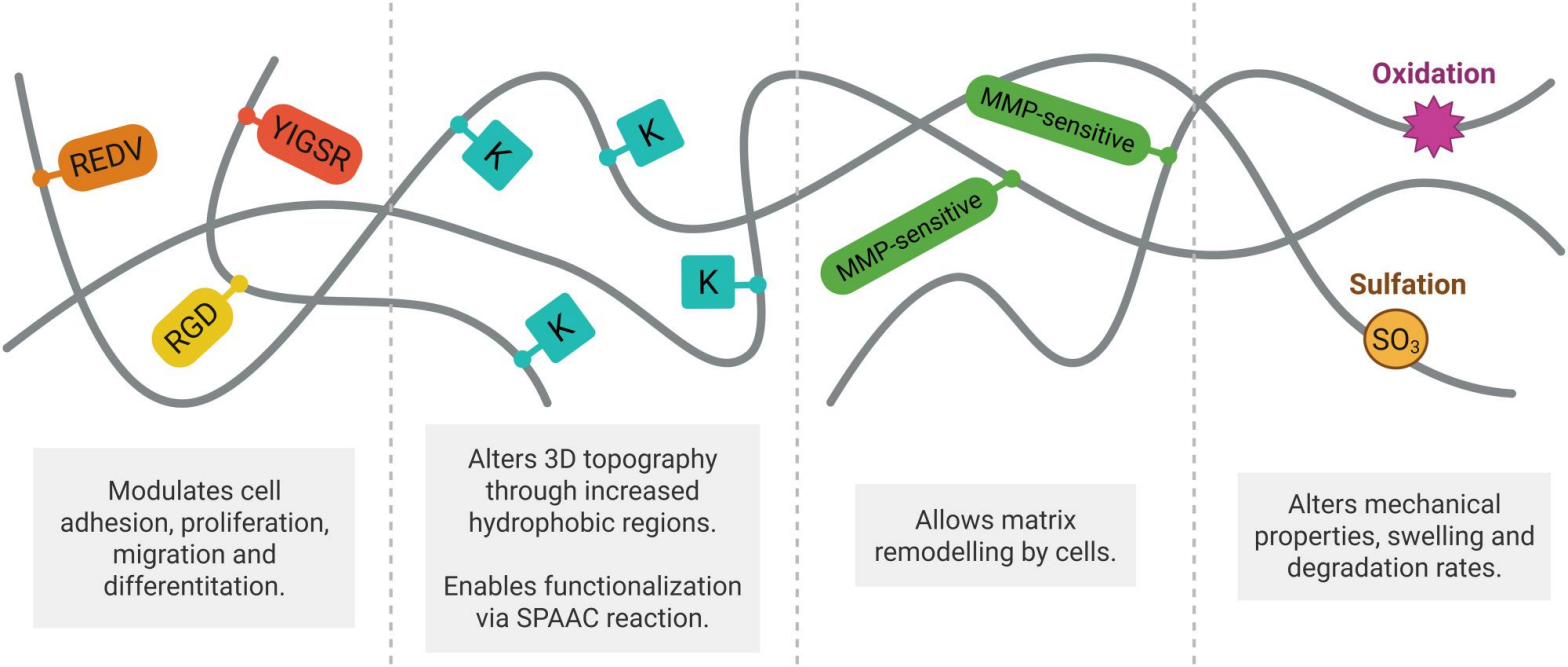
Schematic representation of the most common alginate physicochemical modifications. Created with ‘BioRender.com’. REDV, RGD and YIGSR, amino acid sequences; K, hydrophobic cyclooctyne groups; SPAAC, strain-promoted azide-alkyne cycloaddition; MMP, matrix metalloproteinase.

Alginate is typically used as a hydrogel, a formulation that offers major advantages for wound management due to its ability to maintain adequate moisture, absorb excessive exudate and conform to irregular wound geometries. While these baseline properties already make alginate clinically useful, its lack of inherent cell-adhesive motifs and limited bioactivity constrain its performance in more demanding regenerative contexts. Consequently, chemical functionalization has emerged not merely as an enhancement but as a necessary strategy to transform alginate from a passive dressing into an instructive biomaterial. These modifications can serve very different purposes, ranging from the introduction of specific bioactive moieties to improve cell adhesion, drug retention and controlled release, to the modulation of mechanical properties, degradation rates, crosslinking density, or responsiveness to external stimuli [14, 16, 45]. Given the wide diversity of strategies reported in the literature, the following section highlights only selected representative examples that illustrate the potential of alginate chemical functionalization for wound healing applications. A schematic representation of some alginate chemical functionalization can be found in Figure 2.

Pierschbacher et al. [46] first described the importance of the arginine-glycine-aspartic acid sequence (Arg-Gly-Asp, RGD) for cell attachment and, since then, this small peptide has been widely incorporated into biomaterial-based strategies in a variety of applications [47]. In alginate hydrogels, RGD peptides can be conjugated to the alginate backbone through different techniques, including carbodiimide chemistry [48–50], Cu(I)-catalysed azide–alkyne cycloaddition (CuAAC), strain-promoted azide-alkyne cycloaddition (SPAAC) [51, 52], thiol-ene or thiol-maleimide reactions [53] and reductive amination [54], among others [16, 55]. Compared to unmodified alginate, RGD-functionalized hydrogels demonstrate markedly enhanced cell adhesion, spreading and proliferation. However, more recent studies suggest that single-peptide functionalization may be insufficient to fully recapitulate the complexity of the ECM. In this regard, combinatorial peptide approaches, such as the co-presentation of YIGSR and REDV as described by Yu et al. [56], appear more promising, as they better mimic the multifactorial signalling environment of native tissues and have shown synergistic improvements in angiogenesis and endothelial cell behaviour. These findings indicate that multi-ligand systems may represent a more advanced and biologically relevant direction and recent research on cell-matrix and cell-material interactions resulted in the identification of a variety of bioactive peptides that, when incorporated into biomaterials, modulate cell adhesion and behaviour, including their migration, differentiation and proliferation [57, 58].

Although not designed for wound treatment, Neves et al. [51] described a simple modification of the hydrophilic alginate backbone with hydrophobic cyclooctyne groups (Alg-K), which are highly reactive to azide-functionalized compounds, enabling the hydrogel functionalization with several molecules via SPAAC, a fast and cytocompatible click reaction that can be performed in the presence of cells. Moreover, they showed that the self-association between hydrophobic domains translated into the formation of internal microstructural features, creating a 3D topography that could be sensed by cells, which influenced their morphology and spatial distribution [51]. The same group further demonstrated the functionalization of Alg-K with both RGD peptides and a matrix metalloproteinase (MMP)-sensitive moiety through SPAAC chemistry, in order to promote dermal fibroblasts adhesion to the hydrogel and enable cell-driven enzymatic matrix remodelling, respectively [52]. Compared to Cu(I)-catalysed reactions, SPAAC avoids the use of potentially cytotoxic metal catalysts and offers higher specificity than carbodiimide chemistry, making it especially suitable for *in situ* or cell-encapsulating applications and increasingly viewed as more promising for clinical translation.

For developing tunable biomaterials, Lueckgen et al. [59] oxidized the alginate polymer backbone and subsequently performed covalent crosslinking using norbornene-tetrazine click chemistry. Contrary to ionically crosslinked alginate, which can exhibit poor stability and uncontrolled dissolution, this approach yielded hydrogels with controlled degradation and mechanical properties, which were cell-compatible and susceptible to hydrolytic breakdown, making them suitable for applications requiring gradual material resorption, such as drug delivery or wound healing. In another strategy, Zare-Gachi et al. [60] evaluated the effect of different degrees of sulphation on freeze-dried calcium alginate scaffolds and observed that the highest degree of substitution tested (0.9) resulted in the greatest swelling ratio, although compromising its stability and mechanical integrity. In contrast, scaffolds with an intermediate degree of sulphation (0.5) displayed higher collagen density, a more uniform collagen distribution and lower levels of TGF-β expression, leading to superior outcomes in terms of wound healing in a diabetic mouse model.

These studies highlight the broad potential of alginate chemical functionalization to tailor material properties and introduce specific biological cues. However, enhancing individual functionalities often involves trade-offs that are not always addressed. For example, modifications such as sulphation or oxidation can improve swelling or tunable degradation but may compromise mechanical stability, while increased chemical complexity can introduce toxicity risks, especially with catalysts (e.g. CuAAC) or reactive intermediates that may induce immunogenic responses, compromising the biomaterial’s biocompatibility. Therefore, more cytocompatible strategies, such as SPAAC reactions, should be employed, while a combination of alginate with other biomaterials may improve mechanical properties, as will be discussed later.

It should be noted that the examples described above represent only a small fraction of the wide range of strategies that have been explored to functionalize and engineer alginate for biomedical applications. Beyond peptide conjugation, numerous approaches have been reported to tailor mechanical properties, degradation behaviour, crosslinking mechanisms and responsiveness to environmental cues. Comprehensive discussions of these diverse modification routes, including bioactive ligand incorporation, structural tuning and advanced crosslinking chemistries, can be found in several recent reviews [10, 12, 14, 16, 45, 61].

### Different formulations of alginate for chronic wound therapies

As different diseases and applications require different tissue engineering strategies, another major advantage of alginate is that its use is not restricted to hydrogels, and numerous alternative formulations have been described [6, 62–64]. Compared to standard hydrogels, these alternative formats often address key limitations such as excessive hydrophilicity, limited mechanical strength or lack of multifunctionality. However, their relative effectiveness depends strongly on how well they balance structural complexity with practical applicability.

The gel-forming ability of alginate can be explored in combination with other materials to overcome one of alginate’s main drawbacks, its poor ability to retain hydrophobic drugs. Shafieiuon et al. [65] developed an emulsion gel using dialdehyde carboxymethyl cellulose and CaCO_3_ as crosslinkers via hemiacetal and ionic interactions. These ‘emulgels’ were able to encapsulate curcumin and deliver it into wounds, resulting in accelerated wound closure and reduced inflammation *in vivo*. While promising, their advantage lies primarily in drug encapsulation rather than structural innovation, making them less versatile than more advanced architectures. From a different perspective, Ji et al. [66] employed directional freeze-drying technology and exploited the electrostatic attraction between chitosan and alginate to fabricate an anisotropic aerogel whose aligned channels enabled wound exudate absorption up to 1292.5% of its own weight (Figure 4A), much higher than that of traditional dressings used in the clinic. Despite this high absorbency, the dressing retained favourable mechanical and biological properties, including antioxidant and antibacterial activities, and promoted wound healing in a diabetic mouse model. Lin et al. [67] developed an aerogel based on sodium alginate, chitosan, and calcium peroxide nanoparticles (NPs) capable of continuously releasing oxygen while absorbing wound exudate, thereby addressing hypoxia, which is a major barrier in diabetic wound healing, while simultaneously enhancing collagen deposition and re-epithelialization, contributing to wound repair. Using a distinct approach, da Silva et al. [68] produced a foam dressing composed of calcium alginate and bacterial-derived cellulose incorporating silver-containing graphene oxide NPs, which conferred antimicrobial properties and high exudate absorption capability.

Although gel-like formulations have the advantage of easily adapting to wound topography, in some cases, a more defined structure with stronger mechanical properties may be required. Ding et al. [69] combined polyvinyl alcohol (PVA) with sodium alginate to produce a nanofiber dressing through electrospinning. The dressing was further loaded with shikonin, a natural compound derived from traditional Chinese medicinal plants. This formulation not only exhibited antibacterial and antioxidant properties *in vitro* but also enhanced re-epithelization and wound closure in a mouse model by promoting angiogenesis and reducing inflammation. Electrospinning is an interesting technique for the design of advanced wound healing dressings, since it enables the production of structurally robust dressings that better mimic the fibrous architecture of native ECM. However, it requires the combination of alginate with other polymers, such as PVA, since alginate alone is not readily electrospinnable [70]. Using a different strategy, Alakija et al. [71] produced a stretchable patch for chronic wound treatment based on sodium alginate hydrogel combined with a nanocomposite containing halloysite nanotubes coated with magnesium oxide and silicon nitride. This formulation was extensively characterized and demonstrated the ability to enhance fibroblast migration and proliferation, reduce bacterial activity and maintain high swelling capacity. These stretchable nanocomposite patches introduce additional functionality, such as mechanical resilience and enhanced cell stimulation, but their increased complexity may complicate large-scale production. Hou et al. [72] built a microneedle patch consisting of a needle-like drug-loading gel based on a sodium alginate and polyethylene glycol (PEG) bilayer. These microneedles were able to penetrate bacterial biofilms and effectively release bioactive molecules such as erythromycin, vaccarin and demethylsuberosin, which provided antibacterial and antioxidant effects, while also promoting angiogenesis and reducing inflammation. In addition, the incorporation of cyanidin, a pH-responsive pigment, enabled the patch to visually indicate the wound status according to pH, which directly correlates with infection levels. This microneedle patch stands out as a particularly innovative strategy, since, unlike conventional dressings, it combines both the active delivery of therapeutic agents directly in the wound bed and the diagnostic dimension of real-time monitoring of wound status.

Multilayer dressings represent another promising approach for treating chronic wounds, as they can better mimic the skin’s layered structure and provide multifunctionality. Notably, Latiyan et al. [73] designed a fibro-porous bilayer dressing composed of a lyophilized bottom layer of alginate, agarose and PVA, and an electrospun top layer of curdlan, agarose and PVA. Ciprofloxacin, a broad-spectrum antibiotic, was loaded in both layers, and the dressing demonstrated antibacterial activity, along with high swelling capacity, hemocompatibility and immunomodulatory effects. Moreover, the dressing exhibited minimal fibroblast adhesion, contributing to pain-free dressing changes. Ribeiro et al. [74] developed a three-layer dressing consisting of an outer layer of fibrous polycaprolactone (PCL) to protect the wound from microorganisms, an intermediate sodium alginate layer incorporating ampicillin, and an inner PCL/PEG layer designed to prevent wound adhesion and facilitate dressing exchange. This sandwich-like biomaterial showed mechanical integrity over time, high amounts of exudate absorption and controlled antibiotic release, resulting in antibacterial activity *in vitro*. Nevertheless, further *in vitro* and *in vivo* studies are still required to fully confirm its potential for wound treatment. Compared to single-layer systems, bilayer and trilayer constructs enable the spatial separation of functions, such as antimicrobial protection, exudate absorption and tissue interface optimization. However, despite their conceptual strength, their complexity may hinder translation unless manufacturing processes are streamlined.

An alternative to planar architectures is the development of micro- and nanoscale systems, such as core–shell microparticles. Xu et al. [75] employed all-aqueous microfluidics to generate multifunctional microparticles composed of a calcium alginate and cellulose nanocrystals core loaded with epidermal growth factor (EGF), enclosed by a shell of alkylated chitosan, alginate and ciprofloxacin (Figure 4E). This design enabled the controlled, on-demand release of both the growth factor and the antibiotic, thereby supporting the wound healing process, while presenting antibacterial activity. Nevertheless, its clinical implementation may be limited by challenges in delivery and retention at the wound site.

From a different angle, clay minerals have long been used in wound management due to their ability to stop bleeding and induce anti-inflammatory environments, along with their capacity to promote haemostasis, create an anti-inflammatory microenvironment and degrade into non-toxic products [76]. Since their combination with polymers often results in enhanced mechanical properties, Nomicisio et al. [77] created microspheres composed of alginate, chondroitin sulphate and layered double hydroxide (LDH), a synthetic clay with tunable chemical composition that can be gradually degraded through hydrolysis under acidic conditions, releasing its metal cations. Therefore, in this study, a zinc (Zn) and aluminium (Al)-based LDH was employed, which not only increased the stability of the microspheres in aqueous environments but also enabled the sustained release of bioactive Zn^2+^ ions into the wound. The authors reported enhanced cell proliferation *in vitro* and demonstrated the safety and efficacy of the biomaterial *in vivo*. Also focusing on metal ions, Saadatidizaji et al. [78] developed alginate hydrogel-based beads carrying curcumin, a molecule with anti-inflammatory properties, tetracycline hydrochloride, a widely used antibiotic and CuFe_2_O_4_ NPs to enable the release of Cu^2+^ cations, which exhibit antibacterial activity and pro-angiogenic effects. These nanocomposites proved to be pH- and temperature-responsive, with maximal release occurring at alkaline pH, commonly observed in chronic wounds and at higher temperatures, typically associated with infection [79].

Seeking more practical and minimally invasive formulations for clinic use, Liu et al. [80] developed a sprayable hydrogel composed of alginate and chitosan, in which the *in situ* generation of CO_2_ bubbles created a microporous structure. This system proved to be biocompatible, promoted coagulation and contributed to full wound closure *in vivo*. In a similar approach, Zhou et al. [81] produced a sprayable sodium alginate hydrogel that could be combined with a second spray containing a deep eutectic solvent and copper-tannic acid metal-polyphenol network, enabling rapid *in situ* crosslinking and direct application *in vivo*. In three different animal models, namely acute, infected and burn-infected skin wounds, this sprayable hydrogel promoted wound healing while exhibiting strong antibacterial effects. Shen et al. [82] also designed a sprayable low-oxidized sodium alginate grafting ε-polylysine hydrogel loaded with mesenchymal stem cell (MSC)-derived exosomes for topical application at skin graft donor sites to improve healing and prevent infection. By promoting local macrophage polarization toward an M2-like phenotype, the exosomes helped establish an anti-inflammatory environment that supported tissue repair. Compared to pre-formed dressings, sprayable systems allow rapid, minimally invasive application and excellent conformity to irregular wounds, positioning them among the most promising candidates for future clinical use.

**Figure 3:**
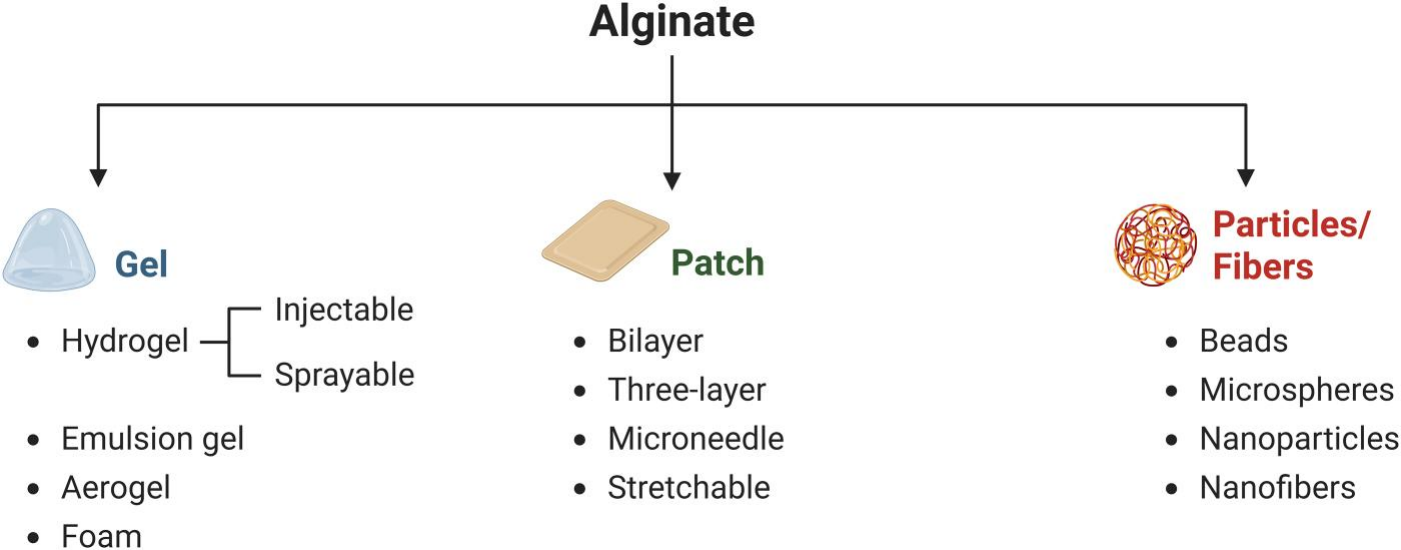
List of the different formulations of alginate. Created with ‘BioRender.com’.

The wide variety of alginate-based formulations, from hydrogels and foams to multilayer and sprayable systems, summarized in Figure 3, demonstrates the material’s adaptability for chronic wound therapeutic needs. While each design targets specific functions such as exudate absorption, oxygen delivery or antimicrobial activity, conjugation of these functionalities and their influence on mechanical performance should be further explored to increase their clinical relevance.

### Combination with other materials

**Figure 4:**
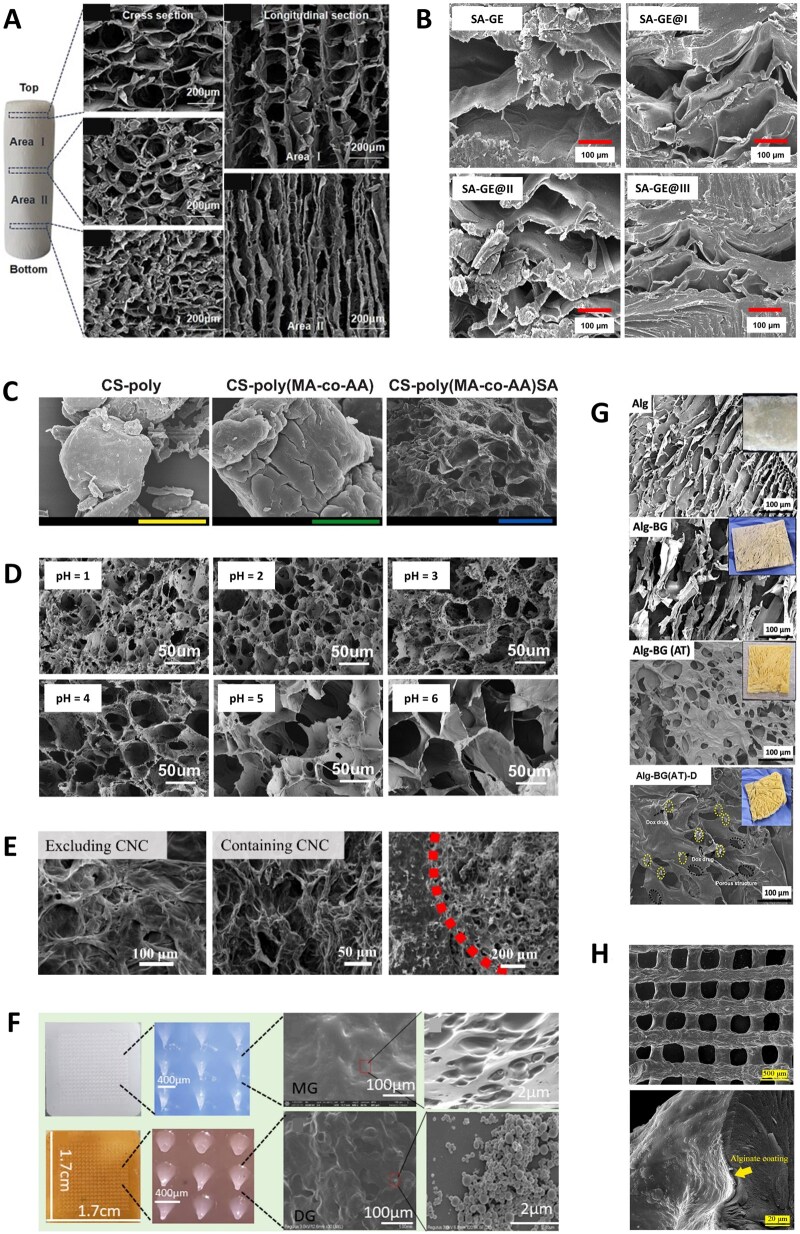
SEM images of combinations of alginate with different biomaterials, reproduced with permission. (**A**) cross and longitudinal directions of different areas of an anisotropic aerogel made of chitosan and sodium alginate (SA) [66]. (**B**) SA and gelatin (GE) hydrogel membranes with varying concentrations of a bimetallic zinc oxide: cerium oxide nanocomposite, namely 1% (SA-GE@I), 2.5% (SA-GE@II), and 5% (SA-GE@III) [90]. (**C**) Chitosan (CS), CS grafted with poly(MA-co-AA), grafted CS+SA [85]. (**D**) A hydrogel made of Fe^3+^, protocatechualdehyde, polyvinyl alcohol (PVA), and SA at different pH values [96]. (**E**) Calcium alginate microparticles with or without cellulose nanocrystals (CNC) and a cross-section of the clear core-shell structure of these microparticles (third image) [75]. (**F**) Matrix gel (MG) and drug-loaded gel (DG) microneedle patches [72]. (**G**) Alginate (Alg) hydrogel membrane incorporated with 45S5 bioglass (Alg-BG), Al_2_O_3_/TiO_2_ nanoparticles (Alg-BG(AT)) and doxycycline (Alg-BG(AT)-D) [98]. (**H**) Morphological and cross-sectional micrographs of a bilayered dressing composed of chitosan loaded with cerium oxide nanoparticles and a vancomycin-loaded alginate coating [89].

Research efforts have also extensively focused on combining alginate with a range of complementary materials to overcome its intrinsic biological and mechanical limitations. For instance, chitosan, a polysaccharide derived from chitin, has long been combined with alginate to elevate the efficacy of natural hydrogel-based wound dressings. Due to their polycationic and polyanionic structures, respectively, chitosan and alginate can form stable polyelectrolyte complexes that improve exudate absorption capacity, drug release efficiency, mechanical properties [83] and antimicrobial activity [84]. For example, this was demonstrated by Raj et al. [85] who combined chitosan and sodium alginate to create a self-healing hydrogel with strong and prolonged antibiofilm activity, confirmed both *in vitro* and *in vivo* (Figure 4C).

Ghodsi et al. [86] used alginate and chitosan to produce hydrogels containing niosomes, non-ionic surfactant-based vesicles structurally similar to liposomes, loaded with curcumin. This system enabled sustained curcumin release, resulting in improved cell viability and enhanced antibacterial properties. In a mouse model, a 90% decrease in wound size was achieved after 10 days, along with an increase in re-epithelization and collagen density and a decrease in inflammation. Specifically, MMP-2, MMP-13 and TGF-β were found to be upregulated, while interleukin (IL)-6, an inflammatory gene, was downregulated. In another approach, Johari et al. [87] investigated the effect of PEG, typically used as a surfactant for copper NPs added to chitosan/sodium alginate hydrogels. The presence of PEG improved degradation behaviour and swelling capacity through the formation of new intra- and intermolecular interactions within the composite, while also contributing to significant antibacterial activity due to a synergistic effect between chitosan and the copper NPs.

Manoharan et al. [88] produced cerium oxide (CeO_2_) NPs that were subsequently encapsulated in a chitosan-alginate hydrogel. The resulting patch was shown to be cytocompatible and to have favourable mechanical properties and swelling capacity, critical features for moisture balance in wounds. Moreover, the cerium oxide conferred antioxidant and antibacterial activity, highlighting its potential for wound healing applications. Shahroudi et al. [89] further advanced this combinatory approach by first 3D-printing a chitosan matrix containing varying concentrations of CeO_2_ NPs, followed by coating the printed scaffold with an alginate layer loaded with vancomycin (Figure 4H). This approach yielded a structured and mechanically robust material based on chitosan, while the alginate coating enhanced NPs release. The dressing demonstrated antibacterial and antioxidant activity *in vitro*, along with confirmed cell biocompatibility. Iqbal et al. [90] also incorporated CeO_2_ NPs into a hydrogel composed of sodium alginate and gelatin instead of chitosan. In this case, CeO_2_ NPs were synthesized in combination with zinc oxide, forming a bimetallic nanocomposite that showed improved antioxidant and antibacterial activity (Figure 4B). While alginate/chitosan hydrogels are robust and relatively simple to implement systems, their functionality is often limited to passive antimicrobial and structural roles unless further modified. In turn, the incorporation of nanocarriers or nanoparticles offers superior control over drug delivery and wound microenvironment modulation, compared to basic polymer blends. Nevertheless, nanoparticle-based approaches raise concerns regarding long-term toxicity, accumulation and regulatory approval, meaning their clinical translation will depend on careful dose optimization.

Alginate and gelatin constitute another attractive combination for wound healing applications. Unlike alginate and chitosan, gelatin inherently provides cell-binding motifs that support cell adhesion, migration and proliferation within the hydrogel, being more effective at promoting re-epithelization and wound closure [91]. Maikovych et al. [92] evaluated hydrogels consisting of alginate and gelatin covalently crosslinked using polyethylene glycol diglycidyl ether (PEGDE 500) and observed increased absorption capacity and enhanced resistance to enzymatic degradation, demonstrating their potential for effective exudate management. Similarly, Puri et al. [93] developed a lyophilized wafer consisting of 2% (w/v) calcium alginate and 1% (w/v) gelatin, which showed outstanding exudate absorption and strong antimicrobial activity *in vitro* due to increased linezolid release. Rizi et al. [94] further explored alginate-gelatin systems by incorporating 10 wt% royal jelly, a natural product rich in bioactive compounds, such as proteins, lipids, flavonoids and phenolic molecules with reported therapeutic potential in chronic wound management. The addition of royal jelly improved hydrogel swelling capacity and provided controlled degradation, while maintaining strong antibacterial activity and promoting tissue regeneration in full-thickness wounds in rats.

Using a different strategy, Li et al. [95] developed an alginate hydrogel modified with aminophenylboronic acid (PBA) and PVA, incorporating tannic acid–ferric ion coordination nanospheres. Through dynamic interactions–including boronic ester bonds, hydrogen bonds and coordination bonds—a multifunctional hydrogel was obtained without the need for additional chemical or physical crosslinking. In a diabetic mouse model of infected full-thickness wounds, the material enhanced wound closure while reducing inflammation and vascular damage. The hydrogel also exhibited photothermal-, pH- and glucose-responsive behaviour. Zeng et al. [96] combined sodium alginate with protocatechualdehyde, PVA and Fe^3+^ to develop a multifunctional hydrogel that was skin-adhesive, self-healing and endowed with anti-inflammatory and antibacterial properties (Figure 4D). Compared to conventional covalently crosslinked hydrogels, these dynamical materials offer responsiveness to pathological conditions, such as infection or hyperglycaemia, being among the most versatile platforms currently available.

Bioactive glasses also offer promising therapeutic potential in wound healing, as they can release biologically active ions that promote angiogenesis, modulate cellular behaviour and provide antibacterial and haemostatic effects [97]. As an example, Bargavi et al. [98] engineered a bioactive nanocomposite hydrogel membrane by combining an alginate hydrogel with a 45S5 bioactive glass containing titania (TiO_2_) and alumina (Al_2_O_3_) NPs (Figure 4G). *In vitro*, this biomaterial promoted collagen and VEGF expression and inhibited biofilm formation, while *in vivo* studies in zebrafish and rat models demonstrated enhanced re-epithelization and accelerated wound closure. Ortiz et al. [99] also included bioglass (54SiO_2_:40CaO:6P_2_O_5_) NPs and zinc oxide (ZnO) NPs in a sodium alginate and PVA mixture that was electrospun into a fibrous membrane. The bioglass NPs were essential to improve the fibres’ mechanical properties, and the final dressing proved to have antimicrobial activity and to be cytocompatible *in vitro*. Bioglass integration into alginate matrices results in materials that are both structurally stable and biologically instructive, but controlling ion release kinetics is critical to avoid cytotoxic effects.

In a related strategy, Marinho et al. [100] used wet-spinning to produce coaxial fibres made of PCL and carbon nanofibers on the outer layer (shell) and sodium alginate loaded with ceftazidime on the inner layer (core) (Figure 4F). The inclusion of carbon nanofibers enhanced the flexibility and resilience of the composite, while also promoting blood clotting, whereas PCL contributed to reduced cytotoxicity. Nazemoroaia et al. [101] designed an asymmetric wound dressing comprising an upper layer of electrospun PCL-silk sericin and a lower chitosan-alginate hydrogel layer loaded with 10-hydroxydecanoic acid (queen bee acid). The hydrogel layer demonstrated high swelling capacity and 80% degradation after 14 days, favourable for early wound healing stages, whereas the top layer showed limited swelling and only 19% degradation, acting as an antibacterial barrier and conferring longer protection to the wound. This dressing was also tested *in vivo* in Wistar rats and demonstrated accelerated wound healing without inflammatory side effects. Using a similar bilayer concept, Gamal et al. [102] 3D-printed a bottom layer of sodium alginate/gelatin hydrogel incorporating pluronics nanomicelles loaded with either rutin, an antioxidant or simvastatin, an anti-inflammatory drug and assembled it with a top layer of electrospun PCL/cellulose acetate nanofibers enriched with chitosan/selenium NPs. This multifunctional bilayer wound dressing showed significant antibacterial activity *in vitro*, while also promoting wound healing *in vivo* in a diabetic rat model, including re-epithelization, collagen deposition, neovascularization and diminished inflammation. As mentioned before, hybrid fibre-based and multilayer systems enable spatial separation of functions, such as mechanical support, drug delivery and antimicrobial protection, also mimicking the hierarchical structure of skin. Therefore, these designs are particularly promising for chronic wounds, as they simultaneously address multiple healing requirements, including infection control, moisture balance and tissue regeneration. However, their increased structural complexity may pose challenges for large-scale manufacturing and cost-effectiveness.

The above-mentioned strategies, summarized in Table 2, combine alginate with complementary materials such as chitosan, gelatin, bioactive glasses or nanoparticles, clearly integrating biological activity, controlled responsiveness and structural organization. In particular, dynamically crosslinked multifunctional hydrogels, bioactive glass-containing composites and multilayer or fibre-based architectures stand out as leading approaches, as they go beyond simple material reinforcement. However, the increasing compositional complexity of these systems raises concerns about reproducibility, scalability and clinical translation.

**Table 2:** Examples of alginate-based composite systems and their main effects on wound treatment.

Composite system	Other biomaterials/molecules incorporated	Main results/effects				Ref.
		Improved mechanical properties	Antimicrobial & antioxidant activity	Increased absorption capacity	Improved wound closure	
Alginate & Chitosan	–	X	X	X	X	[66]
	Calcium peroxide NPs	X	X	X	X	[67]
	–				X	[80]
	–	X	X			[85]
	Curcumin-loaded niosomes		X		X	[86]
	PEG, copper NPs	X	X	X		[87]
	Cerium oxide NPs	X	X	X		[88]
	Cerium oxide NPs, vancomycin		X			[89]
	PCL, silk sericin, 10-hydroxydecanoic acid	X		X	X	[101]
Alginate & Gelatin	Cerium oxide NPs, zinc oxide NPs		X			[90]
	–	X		X		[92]
	Linezolid		X	X		[93]
	Royal jelly	X	X	X	X	[94]
	PCL, cellulose acetate, chitosan, pluronics nanomicelles loaded with rutin or simvastatin, selenium NPs		X		X	[102]
Alginate & PVA	Shikonin		X		X	[69]
	Agarose, curdlan, ciprofloxacin		X	X		[73]
	PBA, tannic acid-ferric ion coordination nanospheres				X	[95]
	Fe^3+^, protocatechualdehyde		X			[96]
	Zinc oxide NPs	X	X			[99]
Alginate & PCL	PEG, ampicillin	X	X	X		[74]
	Carbon nanofibers, ceftazidime	X				[100]

NPs, nanoparticles; PBA, aminophenylboronic acid; PCL, polycaprolactone; PEG, polyethylene glycol; PVA, polyvinyl alcohol.

### Incorporation of bioactive molecules

Chronic wounds present high susceptibility to infection, which often requires prolonged antibiotic administration. Therefore, the incorporation of antibiotics or other antimicrobial compounds into alginate-based dressings offers significant advantages for wound management, even when used as a prophylactic measure. For instance, Zorzi Bueno et al. [103] incorporated the broad-spectrum antibiotic gentamicin in alginate NPs, which were then loaded into chitosan-based films to achieve controlled antibiotic release over time. The incorporation of antibiotic-loaded NPs reduced the swelling of the film, thereby preventing an initial burst release of gentamicin, and providing a more sustained delivery profile compared to the direct incorporation of antibiotic alone. Moreover, drug release was shown to be pH-dependent, resulting in a more effective and responsive release under conditions relevant to infected wounds. Cao et al. [104] employed gelatin and sodium alginate to 3D-print a hydrogel loaded with ciprofloxacin hydrochloride. As predicted, the resultant construct showed outstanding antibacterial activity associated with a fast release of the antibiotic. In terms of wound treatment, the obtained dressing was only tested *in vitro* with a fibroblast cell line (L929) but revealed high biocompatibility and promotion of cell growth and migration. Bibire et al. [105] obtained similar results by incorporating rifampicin, another antibiotic, in alginate grafted with poly(N-vinylcaprolactam).

However, taking into account that overconsumption and misuse of antibiotics have accelerated the development of antibiotic resistance and led to a global health crisis, there is an urgent need to find and implement effective alternative treatments [106]. In this context, Teixeira et al. [107] combined alginate and chitosan to produce a hydrogel loaded with grape pomace extracts. Since these extracts are rich in polyphenols and flavonoids, the formulation exhibited strong antioxidant capacity, along with antibacterial activity, highlighting its potential for wound healing applications. Xu et al. [108] produced poly (vinyl alcohol-co-ethylene) nanofibers and combined them with sodium alginate to produce aerogels that could be either sprayed to form aerogel films or moulded to form aerogel sponges. The film formulation showed high lysozyme adsorption capacity, while the sponge exhibited sustained lysozyme release over time. Given that lysozyme is a potent antimicrobial agent, its incorporation into these aerogel systems enhanced antibacterial performance, besides being non-cytotoxic and having adequate mechanical properties. Birca et al. [109] developed alginate hydrogels containing silver and tannylated calcium peroxide NPs, aiming to combine antimicrobial activity with controlled oxygen release. This approach enabled the mitigation of hypoxia typical of chronic wounds, providing a synergistic effect against bacterial biofilms.

Nevertheless, on-demand strategies should be further explored to enable controlled delivery of antimicrobial molecules according to the specific needs of the wound [110]. To address this, Huang et al. [111] used 3D printing to develop a smart wound dressing based on alginate loaded with calcium phosphate NPs that enabled a pH-responsive degradation and drug release. More precisely, exposure to higher pH media accelerated dressing degradation, which in turn increased rhodamine B, bacitracin, and selenium NPs release, enhancing antimicrobial activity under the alkaline conditions typically found in chronic wounds.

To achieve greater therapeutic efficacy, the incorporation of bioactive molecules into wound dressings should ideally address both antimicrobial activity and the regenerative/healing functions. For example, Da Silva et al. [112] loaded alginate hydrogels with human β-defensin-2, an antimicrobial peptide (AMP), aiming to improve the healing of methicillin-resistant Staphylococcus aureus (MRSA)-infected wounds. In a diabetic mouse model, the peptide reduced MRSA bacterial load and local inflammation, while enhancing cell proliferation and angiogenesis. In a related study, the same group reported a reduction in M1-like macrophages and reactive oxygen species (ROS), together with increased neovascularization and collagen deposition [113]. Bioactive peptides derived from the skin secretions of amphibians, such as RL-QN15, have also been described to accelerate wound healing [114, 115]. The same group that first identified this peptide has since then explored its potential, for example, by loading it inside hollow silica nanoparticles that were then incorporated inside Zn^2+^-crosslinked alginate [116]. The obtained hydrogel offered a slow release of RL-QN15, which resulted in enhanced re-epithelization with controlled angiogenesis and reduced inflammation, while also providing broad-spectrum antimicrobial activity. Compared to traditional antibiotics, these bioactive antimicrobial peptides offer a more holistic approach to wound healing, while reducing the risk of antibiotic resistance.

On another perspective, nitric oxide (NO) plays a dual role as both an antimicrobial agent and a regulator of angiogenesis and inflammation. However, its clinical application is limited by its poor stability and short half-life. To overcome these challenges, Zhang et al. [117] synthesized S-nitrosoglutathione by combining NO with glutathione and subsequently loaded it into a thiolated alginate hydrogel to achieve controlled release. This system enabled a two-phase NO release: an initial high dose burst that enhanced antibacterial activity through bacterial DNA damage and metabolic disruption, followed by a lower sustained NO release that promoted angiogenesis. Similarly, Tan et al. [118] produced an alginate hydrogel loaded with S-nitrosoglutathione using a layer-by-layer melting-permeation crosslinking approach in which one of the layers had a sparser structure to accommodate wound exudates and delay NO release. This hydrogel promoted angiogenesis, accelerated wound closure, and modulated inflammation both *in vitro* and *in vivo*. With comparable results, He et al. [119] encapsulated BNN6, a NO donor, in polymeric NPs and incorporated them in a sodium alginate hydrogel, controlling NO release through near-infrared (NIR) responsive photothermal therapy (PTT).

Decreasing inflammation and promoting neoangiogenesis are also key goals of chronic wound treatments. Wu et al. [120] developed a carboxymethyl chitosan and oxidized sodium alginate-based hydrogel loaded with pH-responsive astilbin liposomes and diclofenac sodium for the treatment of diabetic wounds. The hydrogel’s swelling behaviour correlated with the controlled release of both anti-inflammatory drugs *in vitro* and *in vivo*, leading to antioxidant and antimicrobial effects, reduced inflammation, enhanced angiogenesis and improved fibrotic wound repair. Chai et al. [121] combined sodium alginate, carboxymethyl chitosan and PVA to incorporate taxifolin, an anti-inflammatory drug and HKUST-1, a copper-based metal-organic framework (MOF) that works as a nano-enzyme. Application of this hydrogel in a diabetic mouse model reduced the levels of inflammatory proteins, such as IL-1β, promoted angiogenesis and re-epithelization, and exhibited antibacterial activity *in vitro*. Atia et al. [122] aimed to improve the clinical utility of luteolin, another anti-inflammatory drug with poor solubility and limited skin permeation. To achieve this, luteolin was combined with zein, and the resulting nanocomposite was incorporated into a scaffold composed of hyaluronic acid and sodium alginate. *In vivo* studies in rats demonstrated immune modulation through inhibition of IL-17a secretion and upregulation of IL-13 and VEGF expression. Moreover, miRNA-223 levels were also found to be increased, promoting macrophage polarization into the M2 phenotype. Hosseini et al. [123] created a sodium alginate/carboxymethyl cellulose hydrogel loaded with simvastatin, a drug typically used to reduce lipid levels that has also been associated with angiogenesis promotion and immunomodulation in wound healing. Sustained release of simvastatin for up to 5 days promoted proliferation of fibroblasts and keratinocytes, balanced pro- and anti-inflammatory cytokine profiles *in vitro*, and resulted in faster wound healing in a rat model. Compared to purely antimicrobial systems, these immunomodulatory approaches address the chronic inflammatory state that often impairs healing, being especially promising, as they target underlying pathological mechanisms rather than just symptoms.

In another strategy, Ban et al. [124] produced coacervates of low- and high-molecular-weight cationized gelatin and sodium alginate to encapsulate miR-497, a microRNA that has been previously described by the same authors to exert anti-inflammatory effects in diabetic mice [125]. The coacervates effectively delivered miR-497 to human dermal fibroblasts and keratinocytes, leading to reduced secretion of pro-inflammatory cytokines by the dermal fibroblasts under hyperglycaemic conditions. These findings highlight the potential of this formulation for chronic wound treatment, but further experiments are needed to better characterize its effects *in vivo*. Liu et al. [126] incorporated polyhexamethylene biguanide, an antiseptic polymer, into sodium alginate hydrogels and then loaded them with glargine insulin. This system showed a strong antibacterial effect, while using insulin as a ‘growth factor-like’ agent promoted macrophage polarization into M2, a more anti-inflammatory phenotype. This resulted in decreased levels of IL-1β, IL-6, INF-γ and TNF-α, together with increased expression of the anti-inflammatory cytokine IL-13. Reconstruction of the epidermal basement membrane was also seen *in vivo* through Masson’s, keratin 14 and tight junction ZO-1 staining.

Growth factors are essential regulators of the healing process, and their incorporation in alginate-based formulations can enhance their stability and improve delivery to wound sites [127]. Therefore, Kuan et al. [128] engineered a bilayered alginate hydrogel for growth factor and cytokine delivery. More precisely, polyelectrolyte complex NPs composed of heparan sulphate and chitosan were conjugated with either VEGF, platelet-derived growth factor (PDGF)-BB, or IL-10. Then, the bottom ionically crosslinked alginate layer was loaded with the IL-10 NPs, whereas the top layer of the alginate, consisting of covalently linked gelatin and alginate, was loaded with the VEGF and PDGF-BB NPs. This way, they obtained a multifunctional biomaterial that could first decrease inflammation, due to faster degradation of the bottom layer and consequent release of IL-10, and later increase angiogenesis mediated by gradual delivery of angiogenic growth factors. Karam et al. [129] also designed NP vehicles for growth factor delivery into chronic wounds using PCL combined with either alginate or alginate sulphate, a heparin-mimicking polymer. These NPs proved to be stable, non-toxic and slowly degradable by lysozymes. By loading them with connective tissue growth factor (CTGF) and applying them to full-thickness excisional wounds in mice, they observed that both NP formulations accelerated the healing, demonstrating their potential for wound treatment. Unfortunately, despite the high effectiveness of growth factor delivery systems, their clinical translation is challenged by issues of cost, stability and regulatory approval.

Finally, promoting re-epithelialization is another crucial step for efficient wound closure. With this in mind, Lapmanee et al. [130] incorporated cannabidiol-loaded lipid NPs in a sodium alginate and PVA hydrogel and observed an accelerated wound closure rate in rats, associated with collagen deposition, vascularization and a well-organized epidermal layer. Chen et al. [131] prepared a slow-sculpting hydrogel composed of calcium alginate, graphene oxide and platelet-rich plasma (PRP) that showed high adaptability to irregularly shaped wounds, besides enhancing platelet activity and promoting collagen synthesis and angiogenesis in full-thickness wounds in rats owing to the growth factors present in the PRP. A compilation of the different bioactive molecules embedded in alginate can be found in Table 3.

**Table 3:** Compilation of the different bioactive molecules embedded in alginate.

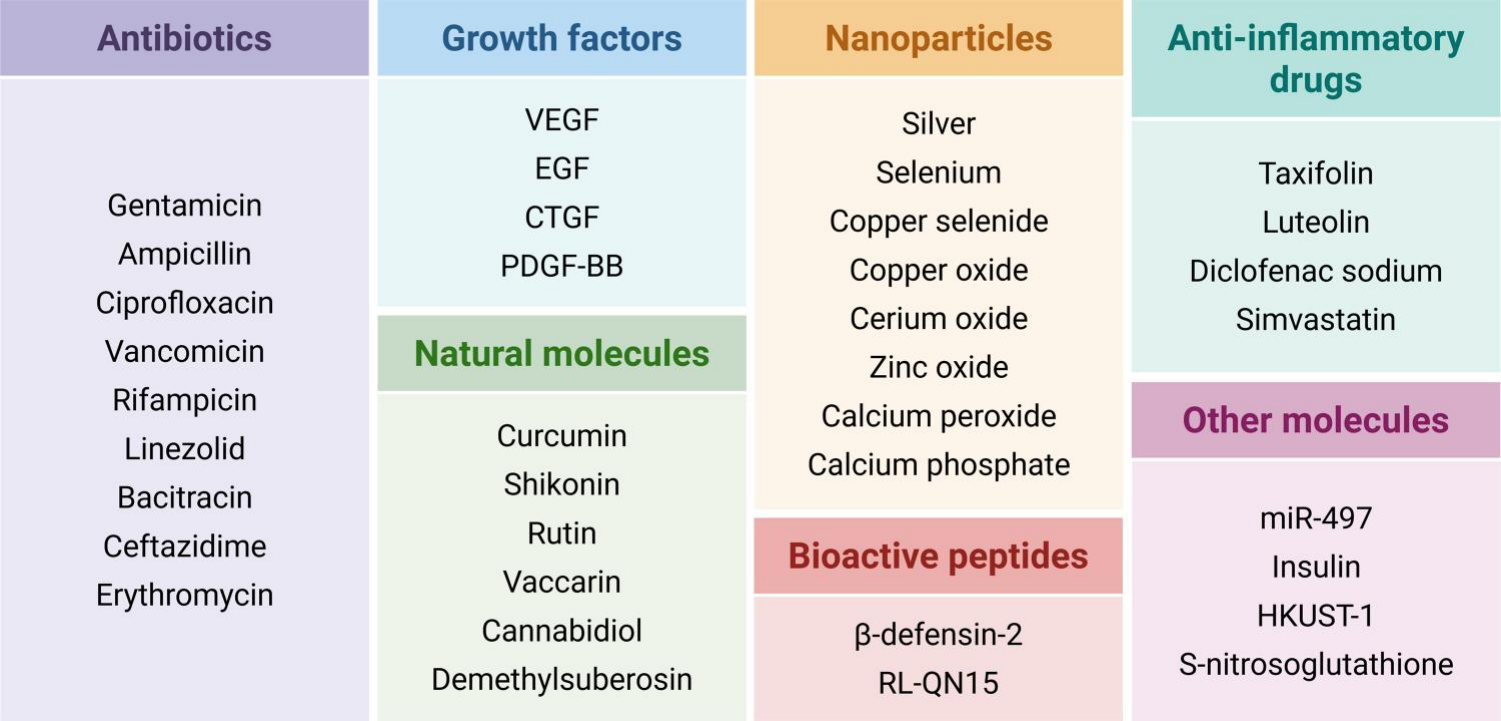

Incorporating bioactive molecules into alginate-based dressings expands therapeutic potential by addressing antimicrobial, anti-inflammatory, angiogenic and regenerative needs. Yet, most studies are limited to short-term or simplified models, making it difficult to predict performance in complex chronic wounds. Moreover, the growing complexity of formulations also challenges reproducibility, manufacturing, and clinical translation. Future work should focus on integrated evaluation of both antimicrobial and regenerative functions under physiologically relevant conditions to ensure that these multifunctional systems provide meaningful and translatable clinical outcomes.

### Incorporation of cells or cell derivatives

Owing to their structural similarity to the ECM of native tissues, alginate hydrogels hold significant potential for cell embedding and delivery to wound sites, as well as for supporting cell migration and proliferation in a 3D environment. These features make alginate-based systems attractive platforms to enhance cell therapy strategies, including for chronic wound treatment [132].

**Figure 5:**
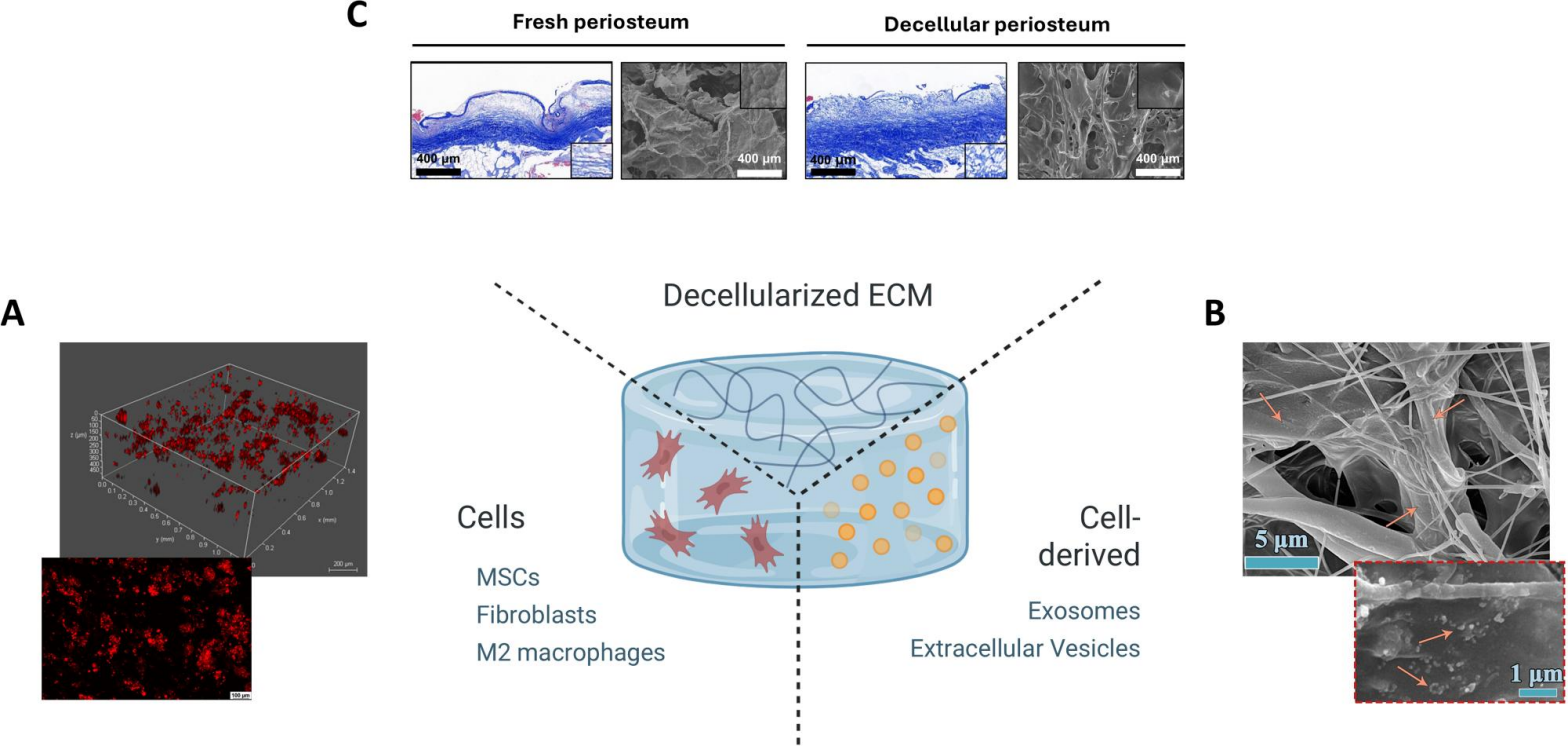
Schematic figure of some of the different cells and cell derivatives incorporated in alginate, reproduced with permission. (**A**) Distribution of M2 macrophages within 2% alginate hydrogel [134]. (**B**) SEM images of exosome-loaded alginate/PCL scaffold. The orange arrows indicate the aggregates of exosomes [153]. (**C**). Masson stains and SEM images of fresh periosteum and decellularized periosteum [157]. created with ‘BioRender.com’.

As discussed earlier, macrophages play a central role in wound healing. Although they exhibit a pro-inflammatory (M1) profile during the early stages, facilitating debris clearance and providing antimicrobial activity, a transition toward an anti-inflammatory (M2) phenotype is required during the repair phase to promote angiogenesis, ECM remodelling and resolution of inflammation. In chronic wounds, however, macrophages frequently remain locked in a persistent M1-dominant state, resulting in excessive inflammation and impaired healing [133]. To address this imbalance, Tian et al. [134] embedded pre-polarized M2 macrophages within alginate hydrogels to modulate macrophage behaviour in the wound bed (Figure 5A), building on previously described concepts [21]. The authors demonstrated that alginate sponges were able to preserve the M2 phenotype, as evidenced by the upregulated expression of anti-inflammatory factors such as IL-10, TGF-β and VEGF. Interestingly, although high expression of both the M2 marker Arg-1 and the M1 marker iNOS was detected, suggesting partial phenotypic plasticity, the macrophage-laden sponges still promoted wound healing in type 2 diabetic mice. Nevertheless, the effectiveness of this strategy is limited by macrophage plasticity, as cells may revert to mixed phenotypes *in vivo*. Therefore, instead of delivering pre-polarized cells, Zhu et al. [135] developed a functional chitosan-based system capable of directing endogenous macrophage polarization into M1 or M2, through modification with either dicyandiamide or PEG. Such an approach could be further enhanced by the incorporation of alginate to improve biomechanical properties and exudate absorption capacity, for example.

Fibroblasts represent another central cellular player in wound repair, as they are the most abundant cells in the dermis and the primary producers of ECM components [136, 137]. With that in mind, Gao et al. [138] developed an injectable alginate hydrogel blended with collagen and fibronectin to improve fibroblast embedding and function. The inclusion of these ECM components greatly increased cell spreading and VEGF secretion *in vitro*, demonstrating the potential of this strategy for wound treatment. In an *in vivo* study, Oushyani Roudsari et al. [139] incorporated mouse embryonic fibroblasts and copper oxide NPs into alginate microgels using a microfluidic platform. This combinatorial approach resulted in faster wound closure due to synergistic effects that enhanced re-epithelialization, angiogenesis and matrix remodelling.

Given their well-known regenerative and immunomodulatory properties, MSCs have also been widely explored for chronic wound applications [140]. MSCs are reported to promote re-epithelialization and angiogenesis, while exerting immunomodulatory and antimicrobial effects, as revised by Nasadiuk et al. [141]. Their accessibility from multiple autologous or allogenic sources, including adipose tissue, dental pulp, Wharton’s jelly, and umbilical cord, and their compatibility with biomaterials and bioactive molecules make MSC-based therapies particularly attractive for chronic wounds [142, 143]. Indeed, MSC therapies have already progressed to clinical evaluation, including in diabetic foot ulcers, where a single application of allogeneic adipose-derived MSCs embedded in a fibrin gel has entered clinical trials (NCT03865394—no results available yet).

Fitriani et al. [144] embedded amniotic membrane stem cells (AMSC), a subtype of MSC, within sodium alginate. The constructs proved to be non-toxic to other cells and achieved complete closure in a scratch assay, although *in vivo* validation remains necessary to fully assess their therapeutic potential. Similarly, Huang et al. [145] incorporated MSCs derived from human perinatal tissues, namely from umbilical cord, chorionic villi, and decidua basalis, into a composite hydrogel of PEGDA, sodium alginate, and collagen I. The authors first characterized the MSCs secretome *in vitro*, identifying enrichment of ECM-remodelling proteins and involvement of the PI3K/AKT signalling pathway, and subsequently confirmed that MSCs-laden hydrogels enhanced re-epithelialization and neovascularization *in vivo*. To further potentiate paracrine mechanisms, Lin et al. [146] conditioned MSCs under hypoxic conditions and applied their secretome to neutrophils *in vitro*, observing suppression of neutrophil extracellular traps (NETs) and ROS production. Building on these findings, hypoxia-conditioned MSCs were encapsulated in an injectable hydrogel composed of sodium alginate and quaternized soy protein isolate grafted with PBA, which enabled on-demand delivery of bioactive factors and resulted in immunomodulation and improved diabetic wound healing *in vivo*.

Despite these promising results, the clinical translation of MSC therapies remains controversial due to variability in isolation protocols and concerns regarding potential malignant transformation [147]. Importantly, many therapeutic effects of MSCs appear to be mediated by secreted paracrine factors rather than direct cell engraftment. These factors are largely packaged within exosomes, a subpopulation of extracellular vesicles (EVs) [148], suggesting that the use of EVs alone may provide safer and more controllable therapeutic alternatives with reduced immunogenicity [149, 150].

In this context, Vakilian et al. [151] isolated MSC-derived EVs and incorporated them into a 3D-printed construct consisting of an alginate core surrounded by a sheath of carboxymethyl cellulose and alginate lyase. The inclusion of the enzyme enabled controlled scaffold degradation, which, in turn, controlled EVs release, while the use of 3D bioprinting provided reproducibility and scalability. Long et al. [152] also incorporated MSC-derived EVs into a sodium alginate hydrogel but combined them with 7A, a 7-amino-acid peptide from histone deacetylase 7 that had previously shown strong angiogenic properties. This combination produced synergistic effects in a diabetic rat model, particularly enhancing fibroblast migration, collagen deposition and angiogenesis. Ashrafi et al. [153] used exosomes derived from human placental MSCs for full-thickness skin wound treatment, but to counteract their rapid clearance, they incorporated them into a bilayer scaffold composed of alginate hydrogel and PCL nanofibers designed to mimic the dermal and epidermal layers of the skin (Figure 5B). This architecture provided sustained exosome release, which in turn promoted collagen synthesis, re-epithelization, and inflammation reduction, ultimately favouring wound closure *in vivo*.

Although alginate hydrogels can support cell proliferation and be combined with bioactive molecules, these strategies only partially recapitulate the complexity of native ECM. An alternative strategy for full tissue regeneration promotion is the use of decellularized matrix (dECM), which preserves native structural and biochemical ECM components, such as collagen fibres, elastin, fibronectin and laminin, in their native architectural structure [154]. This dECM can be blended with alginate and other polymers to improve its biochemical/biophysical properties. For instance, Khosrowpour et al. [155] combined dECM from human placenta with alginate and silk fibroin to fabricate a printable bioink. The resulting 3D constructs supported cell proliferation and, when applied in a mouse model, promoted angiogenesis and re-epithelization without eliciting adverse immune responses. Karmakar et al. [156] produced a hydrogel from amniotic membrane dECM, sodium alginate and carboxymethyl cellulose. This composite proved to have adequate rheological properties, high moisture absorption and improved anti-inflammatory and antibacterial effects. Both Liu et al. [157] and Su et al. [158] developed dressings based on alginate hydrogels combined with periosteum-derived dECM (Figure 5C). While the first authors added nicotinamide mononucleotide to the formulation to boost energy metabolism and reduce oxidative stress, the second group incorporated copper selenide NPs to create a photo-responsive system capable of near-infrared irradiation (NIR)-induced ROS generation for antibacterial purposes. *In vitro*, both strategies promoted angiogenesis and cell proliferation. *In vivo*, the first approach additionally demonstrated anti-inflammatory effects and nerve fibre regeneration, whereas the second focused primarily on antibacterial efficacy.

Alginate-based hydrogels provide a versatile platform for cell delivery and cell-derived factors, supporting angiogenesis, immunomodulation and tissue regeneration, as summarized in Table 4. Nevertheless, despite promising preclinical results, challenges remain in reproducibility, standardization and long-term safety, particularly for MSC-based therapies due to isolation variability and potential malignant transformation. Since many observed therapeutic effects rely on paracrine signalling rather than direct engraftment, cell-free approaches, such as extracellular vesicles, may offer safer and more controllable alternatives. Alternatively, blending decellularized ECM with alginate proved to be a good strategy to improve biochemical and structural mimicry, promoting re-epithelization.

**Table 4:** Summary of the most recent studies incorporating cells and cell derivatives in alginate-based strategies for chronic wound applications.

Biomaterials combined	Cells or cell derivatives incorporated	Main results/effects				Ref.
		Efficient drug release	Improved wound closure	Decreased inflammation	Re-vascularization and re-epithelialization	
Alginate	M2 macrophages		**X**	**X**		[134]
Alginate, collagen, fibronectin	Fibroblasts				**X**	[138]
Alginate, copper oxide NPs	Mouse embryonic fibroblasts		**X**		**X**	[139]
Alginate	Amniotic membrane stem cells		**X**			[144]
Alginate, PEGDA, collagen I	MSCs		**X**		**X**	[145]
Alginate, quaternized soy protein isolate, PBA	Hypoxia-conditioned MSCs			**X**		[146]
Alginate, carboxymethyl cellulose, alginate lyase	MSC-derived EVs	**X**				[151]
Alginate, 7A	MSC-derived EVs		**X**		**X**	[152]
Alginate, PCL nanofibers	MSC-derived exosomes	**X**	**X**	**X**	**X**	[153]
Alginate, silk fibroin	dECM				**X**	[155]
Alginate, carboxymethyl cellulose	dECM			**X**		[156]
Alginate, nicotinamide mononucleotide	dECM			**X**	**X**	[157]
Alginate, copper selenide NPs	dECM				**X**	[158]

dECM, decellularized extracellular matrix; EV, extracellular vesicles; MSC, mesenchymal stem/stromal cells; NPs, nanoparticles; PBA, aminophenylboronic acid; PCL, polycaprolactone; PEGDA, polyethylene glycol diacrylate.

## Future perspectives

### Limitations and challenges of alginate-based wound dressings and how to overcome them

Despite their widespread clinical use and favourable safety profile, commercially available alginate-based wound dressings still exhibit limited capacity to actively modulate the chronic inflammatory environment. Most current-in-use products primarily function as passive exudate absorbers rather than bioactive regulators of wound healing [29, 159]. However, emerging advances demonstrate that alginate hydrogels can be engineered to incorporate anti-inflammatory, immunomodulatory, and regenerative functionalities, originating ‘next generation’ dressings that can actively resolve persistent inflammation and promote progression toward the proliferative phase of healing, addressing a central pathological barrier in chronic wounds.

Although alginate dressings are highly effective in managing exudate, excessive swelling, and limited control over moisture balance can sometimes lead to maceration or hinder cellular infiltration [160]. This highlights the need for more precise tuning of fluid-handling properties. Advances in hydrogel architecture and composite design (e.g. with chitosan or gelatin) allow improved regulation of swelling behaviour and moisture retention, enabling dressings that maintain an optimal moist environment while preserving structural integrity [66, 93]. Such refinements could significantly improve wound bed conditions and support more efficient healing.

Mechanical weakness and structural instability also remain important challenges, particularly in dynamic wound environments such as joints or pressure-prone areas. Unmodified alginate hydrogels are often fragile and lack sufficient tensile strength, which can compromise dressing integrity during use [11]. Advances in material design, including polymer blending and optimized crosslinking strategies, have shown that alginate-based systems can be reinforced to achieve improved mechanical robustness without compromising biocompatibility [161, 162]. Looking forward, the development of mechanically adaptive or stimuli-responsive alginate composites could further enhance durability while maintaining flexibility for complex wound sites.

Another key limitation of clinically used alginate dressings is their insufficient intrinsic antimicrobial activity, which restricts their effectiveness in managing biofilm-associated infections. While current products can absorb exudate and physically entrap microorganisms, they do not inherently prevent bacterial colonization or biofilm persistence [163]. Advanced strategies focus on transforming alginate dressings into active antimicrobial platforms by incorporating bioactive agents such as antibiotics or antimicrobial peptides (e.g. β-defensin-2). These functionalized systems have demonstrated the ability to disrupt biofilms, enhance bacterial clearance and promote tissue repair, highlighting the shift toward infection-responsive therapeutic materials [164]. Nevertheless, traditional alginate systems often exhibit burst release profiles, which may reduce treatment efficacy. To address this, advanced delivery strategies incorporating nanoparticles, microspheres or multi-layered hydrogel systems have been developed to enable sustained and targeted release of bioactive molecules [165]. These innovations provide a pathway toward multifunctional dressings capable of delivering not only antimicrobial drugs, but also anti-inflammatory and pro-regenerative cues, such as cytokines, growth factors and bioactive peptides, over time.

In addition, the lack of intrinsic cell adhesion motifs in alginate limits cellular interaction, which is critical for tissue regeneration and integration [46]. To overcome this limitation, biofunctionalization strategies, like the incorporation of adhesion peptides (e.g. RGD and YIGSR) or ECM components (e.g. collagen and fibronectin), have been explored to enhance cell-material interactions [57]. These approaches offer promising routes to design scaffolds that actively guide cellular behaviour and improve both vascularization and re-epithelialization.

In summary, while commercially available alginate-based dressings already provide essential functions such as exudate management and biocompatibility, their transformation into bioactive and multifunctional systems represents a key future direction. As mentioned throughout this review, several strategies have been employed to address the existing limitations, with good results and potential for clinical application. However, future research should focus on integrating these different strategies to develop multifunctional wound dressings that maximize therapeutic outcomes, while also covering the translational aspects, such as scalability and regulatory approval.

### Future research directions

Although many advanced formulations have demonstrated promising results in preclinical studies, their translation to clinical use remains extremely limited. Consistent with this, there appear to be relatively few ongoing or recently completed clinical trials assessing next-generation alginate-based therapies for chronic wound treatment, with most studies remaining limited to post-market surveillance (e.g. NCT05690685), small feasibility trials or incremental modifications such as silver or honey incorporation (e.g. NCT06873646).

The successful translation of these advanced systems depends on addressing key manufacturing and regulatory challenges. The incorporation of multiple functional components, such as cells, growth factors or nanoparticles, into alginate-based platforms increases production complexity and cost, potentially limiting widespread adoption. At the same time, regulatory frameworks for combination products and bioactive dressings remain complex, requiring rigorous evaluation of long-term safety, stability, efficacy and quality control, and some framework adjustments may be necessary to better accommodate hybrid biomaterial-biological systems [166]. Standardized preclinical models that more accurately mimic chronic wound pathophysiology are needed to generate meaningful and comparable data across studies. In this context, Ojeh et al. [167] proposed ‘The Wound Reporting in Animal and Human Preclinical Studies (WRAHPS) Guidelines’ to improve transparency and consistency across reports and studies before translation into human clinical trials.

Advanced GMP-compatible manufacturing processes should be prioritized early in development to ensure that promising technologies remain feasible for large-scale production and commercialization [168]. Automated 3D printing and continuous fabrication systems can reduce variability and improve reproducibility [89, 155], while integration of artificial intelligence (AI) may contribute to material design and predictive modelling for optimization of alginate formulations. Additionally, AI should be explored for remote wound monitoring in combination with smart dressings that incorporate sensors for oxygen, pH and temperature measurement or even detect bacterial infection [61, 159]. These technologies collectively open the possibility of producing standardized yet customizable wound dressings at scale, bridging the gap between laboratory innovation and real-world clinical application.

Still, it is important to note that the underlying pathophysiology of chronic wounds is still not fully understood, and the heterogeneity of chronic wounds complicates clinical study design, perhaps diluting apparent efficacy across diverse patient populations and limiting the commercial attractiveness and viability despite its therapeutic potential [44, 169]. Therefore, future research should maintain focus on the synergistic integration of different functional components, such as bioactive molecules and composite systems, with technology advances (e.g. AI, 3D-bioprinting), while also dedicating resources to fundamental investigation on chronic wounds pathophysiology, in order to generate multifunctional wound dressings capable of actively modulating the complex wound healing cascade.

## Conclusion

Chronic wounds continue to represent a profound clinical and socioeconomic problem worldwide, affecting millions of patients and contributing substantially to healthcare and expenditure costs due to prolonged treatment, frequent amputations and productivity losses [27]. Moreover, as the global burden of chronic wounds continues to rise with ageing populations and increasing prevalence of diabetes and vascular disease, the need for more effective therapies becomes increasingly urgent. Alginate-based dressings that are commercially available and currently in use in the clinic offer valuable benefits, primarily related to exudate management, haemostasis and provision of a moist healing environment, but fail to address key pathophysiological drivers of chronicity, including persistent inflammation, microbial infection, oxidative stress and impaired tissue repair [2, 23].

Over the past decade, extensive research has focused on converting alginate from a largely passive, absorbent dressing to an active therapeutic platform, changing the paradigm of wound dressings. Multiple strategies have been pursued, ranging from advanced alginate-based formulations, such as nanoparticles, aerogels and microneedles, to composite systems with complementary polymers, together with the incorporation of antimicrobial agents, growth factors, stem cells and cell-derived vesicles. These approaches have shown promising improvements in modulating the wound microenvironment, tissue integration, antimicrobial efficacy, mechanical performance and controlled delivery. Nevertheless, despite excellent *in vitro* and *in vivo* results across many studies, the vast majority of these advanced formulations have not progressed to clinical translation [44]. By integrating advanced design technologies with scalable manufacturing and prioritizing robust clinical validation, cost-effectiveness analyses and clear regulatory strategies, alginate-based biomaterials can move closer to becoming widely adopted, next-generation solutions for chronic wound management. Realizing this potential will require strategic innovation focused on translational feasibility, ultimately enabling advanced alginate-based solutions to move from experimental promise to clinical reality.

## References

[1] Szekalska M , Puciłowska A, Szymańska E, Ciosek P, Winnicka K. Alginate: current use and future perspectives in pharmaceutical and biomedical applications. Int J Polym Sci 2016;2016:1.

[2] Duciel L , Proust R, Ponsen AC, Ziarelli F, Coudreuse A, Jeanmichel L, Samardzic M, Uzan G, des Courtils C. Are all alginate dressings equivalent? J Biomed Mater Res B Appl Biomater 2025;113:e35557. 39976170 10.1002/jbm.b.35557

[3] Orive G , Carcaboso AM, Hernandez RM, Gascon AR, Pedraz JL. Biocompatibility evaluation of different alginates and alginate-based microcapsules. Biomacromolecules 2005;6:927–31. 15762661 10.1021/bm049380x

[4] Alrata L , Abdulsattar D, Madrigal S, Pyeatte SR, Zaghloul M, Abu-Amer W, Arif B, Alhamad T, Remedi M, Lin Y, Zayed MA. Alginate formulation for wound healing applications. Adv Wound Care (New Rochelle) 2025;14:467–78. 39531216 10.1089/wound.2024.0081

[5] Wang Y , Lu Y. Sodium alginate-Based functional materials toward sustainable applications: water treatment and energy storage. Ind Eng Chem Res 2023;62:11279–304.

[6] Sahoo DR , Biswal T. Alginate and its application to tissue engineering. SN Appl Sci 2021;3:30.

[7] Russell Z , Sparling WA, Stewart TL, Gray P, Gaier M, Froning MJ, Mazzanti G, Plucknett KP. Gelation-based feed-stock technologies for HVOF thermal spray development: micro-composite powder preparation and HVOF coating microstructure. Surf Coat Technol 2023;452:129089.

[8] Adamiak K , Sionkowska A. State of innovation in alginate-based materials. Mar Drugs 2023;21:353. 37367678 10.3390/md21060353 PMC10302983

[9] Rahman MM , Shahid MA, Hossain MT, Sheikh MS, Rahman MS, Uddin N, Rahim A, Khan RA, Hossain I. Sources, extractions, and applications of alginate: a review. Discov Appl Sci 2024;6:443.

[10] Li QQ , Xu D, Dong QW, Song XJ, Chen YB, Cui YL. Biomedical potentials of alginate via physical, chemical, and biological modifications. Int J Biol Macromol 2024;277:134409. 39097042 10.1016/j.ijbiomac.2024.134409

[11] Kaklamani G , Cheneler D, Grover LM, Adams MJ, Bowen J. Mechanical properties of alginate hydrogels manufactured using external gelation. J Mech Behav Biomed Mater 2014;36:135–42. 24841676 10.1016/j.jmbbm.2014.04.013

[12] Bidarra SJ , Barrias CC, Granja PL. Injectable alginate hydrogels for cell delivery in tissue engineering. Acta Biomater 2014;10:1646–62. 24334143 10.1016/j.actbio.2013.12.006

[13] Kuo CK , Ma PX. Ionically crosslinked alginate hydrogels as scaffolds for tissue engineering: Part 1. Structure, gelation rate and mechanical properties. Biomaterials 2001;22:511–21. 11219714 10.1016/s0142-9612(00)00201-5

[14] Cao Y , Cong H, Yu B, Shen Y. A review on the synthesis and development of alginate hydrogels for wound therapy. J Mater Chem B 2023;11:2801–29. 36916313 10.1039/d2tb02808e

[15] Wiegand C , Tittelbach J, Hipler U-C, Elsner P. Clinical efficacy of dressings for treatment of heavily exuding chronic wounds. CWCMR 2015;2:101–11.

[16] Neves MI , Moroni L, Barrias CC. Modulating alginate hydrogels for improved biological performance as cellular 3D microenvironments. Front Bioeng Biotechnol 2020;8:665. 32695759 10.3389/fbioe.2020.00665 PMC7338591

[17] Hariyadi DM , Islam N. Current status of alginate in drug delivery. Adv Pharmacol Pharm Sci 2020;2020:8886095. 32832902 10.1155/2020/8886095 PMC7428837

[18] Wallace LA , Gwynne L, Jenkins T. Challenges and opportunities of pH in chronic wounds. Ther Deliv 2019;10:719–35. 31789109 10.4155/tde-2019-0066

[19] Ručigaj A , Kopač T. Mathematical modeling of swelling and shrinking dynamics in pH-sensitive hydrogels composed of alginate, anionic cellulose, and chitosan. Polymer (Guildf) 2025;328:128452.

[20] Sousa AB , Aguas AP, Barbosa MA, Barbosa JN. Immunomodulatory biomaterial-based wound dressings advance the healing of chronic wounds via regulating macrophage behavior. Regen Biomater 2022;9:rbac065. 36267154 10.1093/rb/rbac065 PMC9566965

[21] Gao X , Lu C, Miao Y, Ren J, Cai X. Role of macrophage polarisation in skin wound healing. Int Wound J 2023;20:2551–62. 36785490 10.1111/iwj.14119 PMC10410349

[22] Rodrigues M , Kosaric N, Bonham CA, Gurtner GC. Wound healing: a cellular perspective. Physiol Rev 2019;99:665–706. 30475656 10.1152/physrev.00067.2017 PMC6442927

[23] Falanga V , Isseroff RR, Soulika AM, Romanelli M, Margolis D, Kapp S, Granick M, Harding K. Chronic wounds. Nat Rev Dis Primers 2022;8:50. 35864102 10.1038/s41572-022-00377-3 PMC10352385

[24] Bowers S , Franco E. Chronic wounds: evaluation and management. Am Fam Physician 2020;101:159–66. 32003952

[25] Zhao R , Liang H, Clarke E, Jackson C, Xue M. Inflammation in chronic wounds. Int J Mol Sci 2016;17:2085. 27973441 10.3390/ijms17122085 PMC5187885

[26] James GA , Swogger E, Wolcott R, Pulcini E, Secor P, Sestrich J, Costerton JW, Stewart PS. Biofilms in chronic wounds. Wound Repair Regen 2008;16:37–44. 18086294 10.1111/j.1524-475X.2007.00321.x

[27] Olsson M , Jarbrink K, Divakar U, Bajpai R, Upton Z, Schmidtchen A, Car J. The humanistic and economic burden of chronic wounds: a systematic review. Wound Repair Regen 2019;27:114–25. 30362646 10.1111/wrr.12683

[28] Nowak M , Mehrholz D, Barańska-Rybak W, Nowicki RJ. Wound debridement products and techniques: clinical examples and literature review. Postepy Dermatol Alergol 2022;39:479–90. 35950126 10.5114/ada.2022.117572 PMC9326937

[29] Dabiri G , Damstetter E, Phillips T. Choosing a wound dressing based on common wound characteristics. Adv Wound Care (New Rochelle) 2016;5:32–41. 26858913 10.1089/wound.2014.0586 PMC4717498

[30] Aderibigbe BA , Buyana B. Alginate in wound dressings. Pharmaceutics 2018;10(2):42. 10.3390/pharmaceutics10020042 PMC6027439 29614804

[31] Lou J , Xiang Z, Zhu X, Song J, Huang N, Li J, Jin G, Cui S, Xu P, Le X, Fan Y, Xu S. Evaluating the therapeutic efficacy and safety of alginate-based dressings in burn wound and donor site wound management associated with burn surgery: a systematic review and meta-analysis of contemporary randomized controlled trials. BMC Surg 2025;25:215. 40380141 10.1186/s12893-025-02956-z PMC12083019

[32] O’Meara S , Martyn-St James M, Adderley UJ. Alginate dressings for venous leg ulcers. Cochrane Database Syst Rev 2015;2015:CD010182. 26286189 10.1002/14651858.CD010182.pub3 PMC7087437

[33] Dumville JC , O’Meara S, Deshpande S, Speak K. Alginate dressings for healing diabetic foot ulcers. Cochrane Database Syst Rev 2013;2013:CD009110. 23799857 10.1002/14651858.CD009110.pub3 PMC7111427

[34] Scott Junior WN. Alginate dressings for treating pressure ulcers. Sao Paulo Med J 2015;133:455. 26648438 10.1590/1516-3180.20151335T2 PMC10871798

[35] Paddle-Ledinek JE , Nasa Z, Cleland HJ. Effect of different wound dressings on cell viability and proliferation. Plast Reconstr Surg 2006;117:110S–8S. discussion 119S-120S. 16799377 10.1097/01.prs.0000225439.39352.ce

[36] Terrill P , Sussman G, Bailey M. Absorption of blood by moist wound healing dressings. Primary Intent: Australian J Wound Manag 2003;11:7.

[37] Bonsignore G , Martinotti S, Ranzato E. Wound repair and Ca(2+) signalling interplay: the role of Ca(2+) channels in skin. Cells 2024;13:491. 38534335 10.3390/cells13060491 PMC10969298

[38] Handly LN , Wollman R. Wound-induced Ca(2+) wave propagates through a simple release and diffusion mechanism. Mol Biol Cell 2017;28:1457–66. 28404746 10.1091/mbc.E16-10-0695 PMC5449146

[39] Subramaniam T , Fauzi MB, Lokanathan Y, Law JX. The role of calcium in wound healing. Int J Mol Sci 2021;22:6486. 34204292 10.3390/ijms22126486 PMC8235376

[40] Peltier S , Adib Y, Nicosia L, Ly Ka So S, Da Silva C, Serror K, Duciel L, Proust R, Mimoun M, Bagot M, Bensussan A, des Courtils C, Michel L. In vitro effects of wound-dressings on key wound healing properties of dermal fibroblasts. Exp Dermatol 2024;33:e15098. 38770557 10.1111/exd.15098

[41] Adib Y , Serror K, Pinzon JA, Duciel L, Delagrange M, Ducos B, Boccara D, Mimoun M, Chaouat M, Bensussan A, Samardzic M, Bagot M, Des Courtils C, Michel L. In vitro modulation of macrophage inflammatory and pro-repair properties essential for wound healing by calcium and calcium-alginate dressings. Cells 2025;14:909. 40558536 10.3390/cells14120909 PMC12190423

[42] Wu S , Wu J, Yu H, Zhang J, Huang J, Zhou L, Deng L, Li H. Varying ratios of M/G in alginate to modulate macrophages polarization and its application for wound healing in diabetic. Int J Biol Macromol 2024;270:132387. 38759850 10.1016/j.ijbiomac.2024.132387

[43] Niculescu A-G , Bîrcă AC, Mogoşanu GD, Rădulescu M, Holban AM, Manuc D, Alberts A, Grumezescu AM, Mogoantă L. Zinc alginate hydrogel-coated wound dressings: fabrication, characterization, and evaluation of anti-infective and in vivo performance. Gels 2025;11:427. 40558725 10.3390/gels11060427 PMC12191814

[44] Chen M , Chang C, Levian B, Woodley DT, Li W. Why are there so few FDA-approved therapeutics for wound healing? Int J Mol Sci 2023;24:15109. 37894789 10.3390/ijms242015109 PMC10606455

[45] Hurtado A , Aljabali AAA, Mishra V, Tambuwala MM, Serrano-Aroca A. Alginate: enhancement strategies for advanced applications. Int J Mol Sci 2022;23:4486. 35562876 10.3390/ijms23094486 PMC9102972

[46] Pierschbacher MD , Ruoslahti E. Cell attachment activity of fibronectin can be duplicated by small synthetic fragments of the molecule. Nature 1984;309:30–3. 6325925 10.1038/309030a0

[47] Hersel U , Dahmen C, Kessler H. RGD modified polymers: biomaterials for stimulated cell adhesion and beyond. Biomaterials 2003;24:4385–415. 12922151 10.1016/s0142-9612(03)00343-0

[48] Barros da Silva P , Oliveira RJA, Araujo M, Caires HR, Bidarra SJ, Barrias CC. An integrative alginate-based 3D in vitro model to explore epithelial-stromal cell dynamics in the breast tumor microenvironment. Carbohydr Polym 2024;342:122363. 39048221 10.1016/j.carbpol.2024.122363

[49] Bidarra SJ , Barrias CC, Barbosa MA, Soares R, Granja PL. Immobilization of human mesenchymal stem cells within RGD-grafted alginate microspheres and assessment of their angiogenic potential. Biomacromolecules 2010;11:1956–64. 20690708 10.1021/bm100264a

[50] Fonseca KB , Bidarra SJ, Oliveira MJ, Granja PL, Barrias CC. Molecularly designed alginate hydrogels susceptible to local proteolysis as three-dimensional cellular microenvironments. Acta Biomater 2011;7:1674–82. 21193068 10.1016/j.actbio.2010.12.029

[51] Neves MI , Bidarra SJ, Magalhaes MV, Torres AL, Moroni L, Barrias CC. Microstructured click hydrogels for cell contact guidance in 3D. Mater Today Bio 2023;19:100604. 10.1016/j.mtbio.2023.100604 PMC10034521 36969695

[52] Neves MI , Magalhaes MV, Bidarra SJ, Moroni L, Barrias CC. Versatile click alginate hydrogels with protease-sensitive domains as cell responsive/instructive 3D microenvironments. Carbohydr Polym 2023;320:121226. 37659815 10.1016/j.carbpol.2023.121226

[53] Magalhaes MV , Debera N, Pereira RF, Neves MI, Barrias CC, Bidarra SJ. In situ crosslinkable multi-functional and cell-responsive alginate 3D matrix via thiol-maleimide click chemistry. Carbohydr Polym 2024;337:122144. 38710569 10.1016/j.carbpol.2024.122144

[54] Carvalho ED , Morais MRG, Pego AP, Barrias CC, Araujo M. The interplay between chemical conjugation and biologic performance in the development of alginate-based 3D matrices to mimic neural microenvironments. Carbohydr Polym 2024;323:121412. 37940293 10.1016/j.carbpol.2023.121412

[55] Galindo JM , Merino S, Herrero MA. Advanced hydrogels: enhancing tissue bioengineering with RGD peptides and carbon nanomaterials. ChemMedChem 2025;20:e202400587. 39446356 10.1002/cmdc.202400587 PMC11793852

[56] Yu Y , Guo L, Wang W, Wu J, Yuan Z. Dual-peptide-modified alginate hydrogels for the promotion of angiogenesis. Sci China Chem 2015;58:1866–74.

[57] Hosoyama K , Lazurko C, Munoz M, McTiernan CD, Alarcon EI. Peptide-based functional biomaterials for soft-tissue repair. Front Bioeng Biotechnol 2019;7:205. 31508416 10.3389/fbioe.2019.00205 PMC6716508

[58] Karakaya E , Gleichauf L, Schobel L, Hassan A, Soufivand AA, Tessmar J, Budday S, Boccaccini AR, Detsch R. Engineering peptide-modified alginate-based bioinks with cell-adhesive properties for biofabrication. RSC Adv 2024;14:13769–86. 38681843 10.1039/d3ra08394b PMC11046382

[59] Lueckgen A , Garske DS, Ellinghaus A, Desai RM, Stafford AG, Mooney DJ, Duda GN, Cipitria A. Hydrolytically-degradable click-crosslinked alginate hydrogels. Biomaterials 2018;181:189–98. 30086448 10.1016/j.biomaterials.2018.07.031

[60] Zare-Gachi M , Sadeghi A, Choshali MA, Ghadimi T, Forghani SF, Pezeshki-Modaress M, Daemi H. Degree of sulfation of freeze-dried calcium alginate sulfate scaffolds dramatically influence healing rate of full-thickness diabetic wounds. Int J Biol Macromol 2024;283:137557. 39542337 10.1016/j.ijbiomac.2024.137557

[61] Maity C , Das N. Alginate-based smart materials and their application: recent advances and perspectives. Top Curr Chem (Cham) 2021;380:3. 34812965 10.1007/s41061-021-00360-8

[62] Lai J , Azad AK, Sulaiman W, Kumarasamy V, Subramaniyan V, Alshehade SA. Alginate-based encapsulation fabrication technique for drug delivery: an updated review of particle type, formulation technique, pharmaceutical ingredient, and targeted delivery system. Pharmaceutics 2024;16:370. 38543264 10.3390/pharmaceutics16030370 PMC10975882

[63] Dodero A , Alberti S, Gaggero G, Ferretti M, Botter R, Vicini S, Castellano M. An up-to-date review on alginate nanoparticles and nanofibers for biomedical and pharmaceutical applications. Adv Materials Inter 2021;8:2100809.

[64] Qosim N , Dai Y, Williams GR, Edirisinghe M. Structure, properties, forming, and applications of alginate fibers: a review. Int Mater Rev 2024;69:309–33.

[65] Shafieiuon M , Enayati MH, Ghasemi M, Rostamabadi H. Curcumin delivery via dialdehyde carboxymethyl cellulose/CaCO(3)/GDL cross-linked alginate hydrogels for wound healing. Int J Biol Macromol 2025;321:146183. 40701475 10.1016/j.ijbiomac.2025.146183

[66] Ji M , Li J, Li F, Wang Y, Man J, Wang X, Qiu Y, Zhang C, Peng S, Li J. A double cross-linked anisotropic quaternized chitosan/sodium alginate-based wound dressing for rapid drainage of biofluids. Mater Des 2024;237:112567.

[67] Lin Z , Jia J, Liang C, Ma Y, Liu H, Fang K, Hu Y. Shape-recoverable chitosan/sodium alginate aerogels with sustained oxygen release and antibacterial activity for diabetic wound healing. Int J Biol Macromol 2025;305:141005. 39965687 10.1016/j.ijbiomac.2025.141005

[68] da Silva TF , Ximenes Mesquita J, Luz E, Andrade AL, Kobs H, Teixeira EH, de Souza Filho AG, de Faria AF, Mattos ALA, Andrade FK, Silveira Vieira R. Advanced foam dressing of modified graphene nanoparticles loaded in bacterial cellulose/calcium alginate matrix. ACS Omega 2025;10:28969–81. 40686957 10.1021/acsomega.5c00609 PMC12268464

[69] Ding C , Yang J, Wang N, Ding Q, Sun S, Gao Y, Shen L, Zhao T, Wang Y. Sodium alginate/polyvinyl alcohol nanofibers loaded with shikonin for diabetic wound healing: in vivo and in vitro evaluation. Int J Biol Macromol 2024;262:129937. 38325683 10.1016/j.ijbiomac.2024.129937

[70] Wróbel P , Zwolińska J, Szopa D, Witek-Krowiak A. Towards enhanced electrospinning of Alginate—can recent strategies overcome limitations? A review. Polymers (Basel) 2025;17:2255. 40871202 10.3390/polym17162255 PMC12390050

[71] Alakija FB , Mills DK. Fabrication and characterization of a stretchable sodium alginate hydrogel patch combined with silicon nitride and metalized halloysite nanotubes to develop a chronic wound healing treatment. Int J Mol Sci 2025;26:1734. 40004197 10.3390/ijms26041734 PMC11855668

[72] Hou B , Xu A, Zhang S, Cai W, Wen Y, Wang Y, Zhu X, Huang S, Huang J, Qiu L, Sun H. Application of sodium alginate and polyethylene glycol bilayer multifunctional hydrogel microneedles in infectious and diabetic wounds. Int J Biol Macromol 2025;310:143471. 40288706 10.1016/j.ijbiomac.2025.143471

[73] Latiyan S , Kumar TSS, Doble M. Functionally multifaceted alginate/curdlan/agarose-based bilayer fibro-porous dressings for addressing full-thickness diabetic wounds. Biomater Adv 2024;157:213757. 38198999 10.1016/j.bioadv.2023.213757

[74] Ribeiro ARM , Teixeira MO, Ribeiro L, Tavares TD, Miranda CS, Costa AF, Ribeiro A, Silva MM, Silva C, Felgueiras HP. Sodium alginate-based multifunctional sandwich-like system for treating wound infections. Biomater Adv 2024;162:213931. 38924805 10.1016/j.bioadv.2024.213931

[75] Xu F , Wang W, Zhao W, Zheng H, Xin H, Sun W, Ma Q. All-aqueous microfluidic fabrication of calcium alginate/alkylated chitosan core-shell microparticles with time-sequential functions for promoting whole-stage wound healing. Int J Biol Macromol 2024;282:136685. 39454904 10.1016/j.ijbiomac.2024.136685

[76] Mousa M , Evans ND, Oreffo ROC, Dawson JI. Clay nanoparticles for regenerative medicine and biomaterial design: a review of clay bioactivity. Biomaterials 2018;159:204–14. 29331807 10.1016/j.biomaterials.2017.12.024

[77] Nomicisio C , Ruggeri M, Taviot-Guého C, Boselli C, Icaro Cornaglia A, Bianchi E, Del Favero E, Ricci C, Firpo G, Vigani B, Vacca P, Viseras C, Rossi S, Sandri G. Development of clay/alginate/chondroitin sulfate composite microspheres via continuous process for chronic wounds regeneration. Carbohydr Polym Technol Appl 2025;10:100711.

[78] Saadatidizaji Z , Sohrabi N, Mohammadi R, Amini-Fazl MS. Tetracycline hydrochloride loaded-alginate based nanoparticle-hydrogel beads for potential wound healing applications: in vitro drug delivery, release kinetics, and antibacterial activity. Int J Biol Macromol 2024;264:130653. 38458272 10.1016/j.ijbiomac.2024.130653

[79] Huang J , Fan C, Ma Y, Huang G. Exploring thermal dynamics in wound healing: the impact of temperature and microenvironment. Clin Cosmet Investig Dermatol 2024;17:1251–8. 10.2147/CCID.S468396 PMC11144001 38827629

[80] Liu C , Li J, Shi Z, Wang C, Li J, Wang X, Huang F. Sprayable macroporous alginate/chitosan stacked hydrogels for enhancing wound healing. Int J Biol Macromol 2025;319:145678. 40602576 10.1016/j.ijbiomac.2025.145678

[81] Zhou R , Zhou M, Wang W, Huang J, Peng W, Wang L, Chen J, Bo R, Liu M, Li J. A sprayable alginate hydrogel based on copper-tannic acid metal-polyphenol for promoting acute and infected wound healing. Int J Biol Macromol 2025;321:146323. 40730304 10.1016/j.ijbiomac.2025.146323

[82] Shen Z , Wang L, Xie X, Yuan W. Sprayable, antimicrobial and immunoregulation hydrogel loading exosomes based on oxidized sodium alginate for efficient wound healing at skin graft donor sites and health detection. Carbohydr Polym 2025;351:123098. 39779012 10.1016/j.carbpol.2024.123098

[83] Saberian M , Safari Roudsari R, Haghshenas N, Rousta A, Alizadeh S. How the combination of alginate and chitosan can fabricate a hydrogel with favorable properties for wound healing. Heliyon 2024;10:e32040. 38912439 10.1016/j.heliyon.2024.e32040 PMC11192993

[84] Wathoni N , Suhandi C, Ghassani Purnama MF, Mutmainnah A, Nurbaniyah NS, Syafra DW, Elamin KM. Alginate and chitosan-based hydrogel enhance antibacterial agent activity on topical application. Infect Drug Resist 2024;17:791–805. 38444772 10.2147/IDR.S456403 PMC10913799

[85] Raj V , Raorane CJ, Shastri D, Kim SC, Lee S. Engineering a self-healing grafted chitosan-sodium alginate based hydrogel with potential keratinocyte cell migration property and inhibitory effect against fluconazole resistance *Candida albicans* biofilm. Int J Biol Macromol 2024;261:129774. 38286383 10.1016/j.ijbiomac.2024.129774

[86] Ghodsi R , Sadr Tahouri SK, Amjad F, Nasiri MR, Toutounchi A, Salehi H, Moeinzadeh A, Sadeghi S, Soleimani N, Onagh B, Farmani AR, Tavakkoli Yaraki M. Chitosan/alginate@niosome-curcumin: high-performance wound dressing with enhanced antibacterial activity. J Drug Deliv Sci Technol 2025;114:107438.

[87] Johari N , Rahimi F, Azami H, Rafati F, Nokhbedehghan Z, Samadikuchaksaraei A, Moroni L. The impact of copper nanoparticles surfactant on the structural and biological properties of chitosan/sodium alginate wound dressings. Biomater Adv 2024;162:213918. 38880016 10.1016/j.bioadv.2024.213918

[88] Manoharan S , Balakrishnan P, Sellappan LK, Sanmugam A. Green synthesized cerium oxide nanoparticles incorporated chitosan-alginate based nanobiopatch for enhanced antibacterial wound dressing applications. J Drug Deliv Sci Technol 2025;108:106892.

[89] Shahroudi S , Parvinnasab A, Salahinejad E, Abdi S, Rajabi S, Tayebi L. Efficacy of 3D-printed chitosan-cerium oxide dressings coated with vancomycin-loaded alginate for chronic wounds management. Carbohydr Polym 2025;349:123036. 39638529 10.1016/j.carbpol.2024.123036

[90] Iqbal Y , Amin F, Aziz MH, Khalid M, Alhadlaq HA, Alaizeri ZM. Flexible sodium alginate-gelatin hydrogel membrane incorporated with green synthesized bimetallic ZnO: ceO2 nanocomposite for antioxidant, antibacterial and biocompatibility studies. React Funct Polym 2025;212:106228.

[91] Ndlovu SP , Ngece K, Alven S, Aderibigbe BA. Gelatin-based hybrid scaffolds: promising wound dressings. Polymers (Basel) 2021;13:2959. 34502997 10.3390/polym13172959 PMC8434607

[92] Maikovych O , Pasetto P, Nosova N, Kudina O, Ostapiv D, Samaryk V, Varvarenko S. Functional properties of gelatin-alginate hydrogels for use in chronic wound healing applications. Gels 2025;11:174. 40136880 10.3390/gels11030174 PMC11941921

[93] Puri GK , Jania RK, Upadhye V, Sharma MC, Hassan SMS, Alsaidan OA, Hajare ST. Calcium alginate medicated dressing for enhanced absorption in diabetic foot ulcer management. Sci Rep 2025;15:23335. 40604094 10.1038/s41598-025-05982-2 PMC12222941

[94] Rizi EM , Ebrahimian-Hosseinabadi M, Kharazi AZ. Royal jelly-enriched alginate/gelatin hydrogel film for effective treatment of chronic skin wounds. Mater Today Commun 2025;46:112859.

[95] Li D , Li M, Wang L, Zhang J, Wang X, Nie J, Ma G. The synergetic effect of alginate-derived hydrogels and metal-phenolic nanospheres for chronic wound therapy. J Mater Chem B 2024;12:2571–86. 38363109 10.1039/d3tb02685j

[96] Zeng H , Tang L, Huang L, Yang N, Chen X, Peng X, Chen Z, Guo J, Weng J, Guo T. A novel multi-functional PVA-alginate hydrogel with dynamic bond crosslinking for infected wound repair. Carbohydr Polym 2025;362:123636. 40409832 10.1016/j.carbpol.2025.123636

[97] Pawar V , Shinde V. Bioglass and hybrid bioactive material: a review on the fabrication, therapeutic potential and applications in wound healing. Hybrid Adv 2024;6:100196.

[98] Bargavi P , Balakumar S, Raghunandhakumar S. Multi-functional bandage - bioactive glass/metal oxides/alginate composites based regenerative membrane facilitating re-epithelialization in diabetic wounds with sustained drug delivery and anti-bactericidal efficacy. Int J Biol Macromol 2024;262:130054. 38342258 10.1016/j.ijbiomac.2024.130054

[99] Ortiz JA , Sepulveda FA, Flores S, Saavedra M, Saez-Silva S, Jimenez T, Murgas P, Troncoso S, Sanhueza C, Ulloa MT, Porte Torre L, Ahumada M, Corrales T, Palza H, Zapata PA. Electrospun polyvinyl alcohol/sodium alginate nanocomposite dressings loaded with ZnO and bioglass: characterization, antibacterial activity, and cytocompatibility. Polymers (Basel) 2025;17:2185. 40871132 10.3390/polym17162185 PMC12388954

[100] Marinho E , Silva BM, Miranda CS, Pinho SLC, Felgueiras HP. Polycaprolactone/sodium alginate coaxial wet-spun fibers modified with carbon nanofibers and ceftazidime for improved clotting and infection control in wounds. Biomater Sci 2025;13:2047–65. 40026077 10.1039/d4bm01667j

[101] Nazemoroaia M , Bagheri F, Mirahmadi-Zare SZ, Eslami-Kaliji F, Derakhshan A. Asymmetric natural wound dressing based on porous chitosan-alginate hydrogel/electrospun PCL-silk sericin loaded by 10-HDA for skin wound healing: in vitro and in vivo studies. Int J Pharm 2025;668:124976. 39577507 10.1016/j.ijpharm.2024.124976

[102] Gamal AA , Yehia S, Sayed HAE, Hussein MAM, El-Sayed EM, El-Sherbiny IM. Multifunctional bilayer wound dressing based on 3D-printed sodium alginate/gelatin hydrogel and polycaprolactone nanofibers for accelerating diabetic wound healing. Int J Biol Macromol 2025;318:144832. 40466840 10.1016/j.ijbiomac.2025.144832

[103] Zorzi Bueno C , Wiggers HJ, Chevallier P, Copes F, Mantovani D. Chitosan films loaded with alginate nanoparticles for gentamicin release on demand. Polymers (Basel) 2025;17:2261. 40871208 10.3390/polym17162261 PMC12390268

[104] Cao L , Lu Y, Chen H, Su Y, Cheng Y, Xu J, Sun H, Song K. A 3D bioprinted antibacterial hydrogel dressing of gelatin/sodium alginate loaded with ciprofloxacin hydrochloride. Biotechnol J 2024;19:e2400209. 39212214 10.1002/biot.202400209

[105] Bibire T , Dănilă R, Yilmaz CN, Verestiuc L, Nacu I, Ursu RG, Ghiciuc CM. In vitro biological evaluation of an alginate-based hydrogel loaded with rifampicin for wound care. Pharmaceuticals (Basel) 2024;17:943. 39065793 10.3390/ph17070943 PMC11280071

[106] Aggarwal R , Mahajan P, Pandiya S, Bajaj A, Verma SK, Yadav P, Kharat AS, Khan AU, Dua M, Johri AK. Antibiotic resistance: a global crisis, problems and solutions. Crit Rev Microbiol 2024;50:896–921. 38381581 10.1080/1040841X.2024.2313024

[107] Teixeira LS , Sousa M, Massano F, Borges A. Exploring grape pomace extracts for the formulation of new bioactive multifunctional chitosan/alginate-based hydrogels for wound healing applications. Food Biosci 2024;62:105073.

[108] Xu J , Wu Q, Wang J, Liu Y, Liu K, Xia M, Wang D. Advanced alginate-based nanofiber aerogels: a synthetic matrix for high-efficiency lysozyme adsorption and controlled release. Int J Biol Macromol 2024;280:135974. 39332565 10.1016/j.ijbiomac.2024.135974

[109] Bîrcă AC , Gherasim O, Niculescu A-G, Grumezescu AM, Vasile BȘ, Mihaiescu DE, Neacșu IA, Andronescu E, Trușcă R, Holban AM, Hudiță A, Croitoru G-A. Infection-Free and enhanced wound healing potential of alginate gels incorporating silver and tannylated calcium peroxide nanoparticles. IJMS 2024;25:5196. 38791232 10.3390/ijms25105196 PMC11120750

[110] Das IJ , Bal T. pH factors in chronic wound and pH-responsive polysaccharide-based hydrogel dressings. Int J Biol Macromol 2024;279:135118. 39208902 10.1016/j.ijbiomac.2024.135118

[111] Huang T , Sun Z, Heath DE, O’Brien-Simpson N, O’Connor AJ. 3D printed and smart alginate wound dressings with pH-responsive drug and nanoparticle release. Chem Eng J 2024;492:152117.

[112] Da Silva J , Calheiros D, Goncalves T, Silva EA, Carvalho E, Leal EC. Alginate-based hydrogels loaded with human beta-defensin-2 promote healing of MRSA-infected wounds in a diabetic model: a preclinical proof-of-concept study. Clin Exp Med 2025;25:250. 40663184 10.1007/s10238-025-01798-6 PMC12263799

[113] Da Silva J , Leal EC, Gomes A, Gomes P, Calheiros D, Goncalves T, Carvalho E, Silva EA. Alginate-based hydrogels for sustained antimicrobial peptide delivery to enhance wound healing in diabetes. Biomater Adv 2025;175:214337. 40359773 10.1016/j.bioadv.2025.214337

[114] Wang Y , Feng Z, Yang M, Zeng L, Qi B, Yin S, Li B, Li Y, Fu Z, Shu L, Fu C, Qin P, Meng Y, Li X, Yang Y, Tang J, Yang X. Discovery of a novel short peptide with efficacy in accelerating the healing of skin wounds. Pharmacol Res 2021;163:105296. 33220421 10.1016/j.phrs.2020.105296

[115] Zheng K , Yang Z, Ba T. Marine bioactive peptides as potential therapeutic agents for wound healing - a review. Ann Med 2025;57:2530693. 40652403 10.1080/07853890.2025.2530693 PMC12258173

[116] Qin P , Tang J, Sun D, Yang Y, Liu N, Li Y, Fu Z, Wang Y, Li C, Li X, Zhang Y, Liu Y, Wang S, Sun J, Deng Z, He L, Wang Y, Yang X. Zn(2+) Cross-Linked alginate carrying hollow silica nanoparticles loaded with RL-QN15 peptides provides promising treatment for chronic skin wounds. ACS Appl Mater Interfaces 2022;14:29491–505. 35731847 10.1021/acsami.2c03583

[117] Zhang Y , Tan J, Tu X, Tan D, Yu L, Xie Y, Li N, Zhang S, Li L, Zhou C. Rapid formulation alginate dressing with sustained nitric oxide release: a dual-action approach for antimicrobial and angiogenic benefits. Biomater Adv 2025;176:214350. 40414082 10.1016/j.bioadv.2025.214350

[118] Tan J , Wen M, Zhang Y, Zhang S, Fang M, Xiang J, Liu X, Tian J, Lu L, Luo B, Zhou C, Li L. Development of an asymmetric alginate hydrogel loaded with S-Nitrosoglutathione and its application in chronic wound healing. Gels 2025;11:354. 40422374 10.3390/gels11050354 PMC12111571

[119] He C , Bi S, Zhang L, Gu J, Yan B. An antioxidative sodium alginate hybrid hydrogel with NIR-controlled NO releasing for diabetic wound healing via reduced inflammation and enhanced angiogenesis. Carbohydr Polym 2025;366:123913. 40733834 10.1016/j.carbpol.2025.123913

[120] Wu X , Wang Y, Liu X, Ding Q, Zhang S, Wang Y, Chai G, Tang Y, Yang J, Yu T, Liu W, Ding C. Carboxymethyl chitosan and sodium alginate oxide pH-sensitive dual-release hydrogel for diabetes wound healing: the combination of astilbin liposomes and diclofenac sodium. Carbohydr Polym 2025;349:122960. 39638514 10.1016/j.carbpol.2024.122960

[121] Chai G , Wang N, Xu M, Ma L, Liu X, Ding Q, Zhang S, Li A, Xia G, Zhao Y, Liu W, Liang D, Ding C. Poly (vinyl alcohol)/sodium alginate/carboxymethyl chitosan multifunctional hydrogel loading HKUST-1 nanoenzymes for diabetic wound healing. Int J Biol Macromol 2024;268:131670. 38643919 10.1016/j.ijbiomac.2024.131670

[122] Atia NM , Shahine YM, Abdallah OY, Ghany MSA, Moustafa MA. Novel Luteolin-Zein nanocomposite incorporated in hyaluronic acid/sodium alginate scaffold as potential immunomodulation for pressure ulcer wounds. AAPS PharmSciTech 2025;26:185. 40613860 10.1208/s12249-025-03181-w

[123] Hosseini SMR , Heydari P, Namnabat M, Nasr Azadani R, Azimi Gharibdousti F, Mousavi Rizi E, Khosravi A, Zarepour A, Zarrabi A. Carboxymethyl cellulose/sodium alginate hydrogel with anti-inflammatory capabilities for accelerated wound healing: In vitro and in vivo study. Eur J Pharmacol 2024;976:176671. 38797311 10.1016/j.ejphar.2024.176671

[124] Ban E , Park M, Kim Y, Park J, Kim A. Enhanced delivery of anti-inflammatory miRNA-497 to dermal fibroblasts using cationized gelatin-sodium alginate coacervates. J Drug Deliv Sci Technol 2024;97:105767.

[125] Ban E , Jeong S, Park M, Kwon H, Park J, Song EJ, Kim A. Accelerated wound healing in diabetic mice by miRNA-497 and its anti-inflammatory activity. Biomed Pharmacother 2020;121:109613. 31707336 10.1016/j.biopha.2019.109613

[126] Liu D , Yu T, Ma S, Su L, Zhong S, Wang W, Liu Y, Yu JA, Gao M, Chen Y, Xu H, Liu Y. Insulin/PHMB-grafted sodium alginate hydrogels improve infected wound healing by antibacterial-prompted macrophage inflammatory regulation. J Nanobiotechnol 2025;23:328. 10.1186/s12951-025-03398-8 PMC12048987 40319298

[127] Wawrzyńska E , Kubies D. Alginate matrices for protein delivery—a short review. Physiol Res 2018;67:S319-34. 30379553 10.33549/physiolres.933980

[128] Kuan CH , Chang L, Ho CY, Tsai CH, Liu YC, Huang WY, Wang YN, Wang WH, Wang TW. Immunomodulatory hydrogel orchestrates pro-regenerative response of macrophages and angiogenesis for chronic wound healing. Biomaterials 2025;314:122848. 39342917 10.1016/j.biomaterials.2024.122848

[129] Karam M , Faraj M, Jaffa MA, Jelwan J, Aldeen KS, Hassan N, Mhanna R, Jaffa AA. Development of alginate and alginate sulfate/polycaprolactone nanoparticles for growth factor delivery in wound healing therapy. Biomed Pharmacother 2024;175:116750. 38749174 10.1016/j.biopha.2024.116750

[130] Lapmanee S , Bhubhanil S, Charoenphon N, Inchan A, Bunwatcharaphansakun P, Khongkow M, Namdee K. Cannabidiol-Loaded lipid nanoparticles incorporated in polyvinyl alcohol and sodium alginate hydrogel scaffold for enhancing cell migration and accelerating wound healing. Gels 2024;10:843. 39727600 10.3390/gels10120843 PMC11675964

[131] Chen N , Li M, Yang J, Wang P, Song G, Wang H. Slow-sculpting graphene oxide/alginate gel loaded with platelet-rich plasma to promote wound healing in rats. Front Bioeng Biotechnol 2024;12:1334087. 38390356 10.3389/fbioe.2024.1334087 PMC10882075

[132] Kavand A , Noverraz F, Gerber-Lemaire S. Recent advances in Alginate-Based hydrogels for cell transplantation applications. Pharmaceutics 2024;16:469. 38675129 10.3390/pharmaceutics16040469 PMC11053880

[133] Krzyszczyk P , Schloss R, Palmer A, Berthiaume F. The role of macrophages in acute and chronic wound healing and interventions to promote pro-wound healing phenotypes. Front Physiol 2018;9:419. 29765329 10.3389/fphys.2018.00419 PMC5938667

[134] Tian Q , Li W, Zhang L, Gan K, Zhang Y, Wang S. Sodium alginate hydrogel sponges embedded with M2 macrophages: an adoptive cell therapy strategy for accelerated diabetic wound healing. Gels 2025;11:502. 40710665 10.3390/gels11070502 PMC12294235

[135] Zhu M , Ou J, Chen Y, Tian Y, Song W, Hu X, Ju X, Jiang S, Huang S, Niu Z. Programming of macrophage polarization in different stages for accelerating wound healing. Chem EngJ 2024;491:152131.

[136] Graham HK , Eckersley A, Ozols M, Mellody KT, Sherratt MJ. Human skin: composition, structure and visualisation methods. In: Limbert G (ed). Skin Biophysics: From Experimental Characterisation to Advanced Modelling. Cham: Springer International Publishing, 2019,1-18.

[137] Boraldi F , Lofaro FD, Bonacorsi S, Mazzilli A, Garcia-Fernandez M, Quaglino D. The role of fibroblasts in skin homeostasis and repair. Biomedicines 2024;12:1586. 39062158 10.3390/biomedicines12071586 PMC11274439

[138] Gao D , Shipman WD, Sun Y, Yang W, Mathew AT, Beraki L, Glahn JZ, Kochen A, Kyriakides TR, Horsley V, Hsia HC. An injectable alginate hydrogel modified by collagen and fibronectin for better cellular environment. ACS Appl Bio Mater 2025;8:1675–83. 10.1021/acsabm.4c01853 39886738

[139] Oushyani Roudsari Z , Nedaei K, Araghi M, Mortazavi Y, Nadri S. Wound tissue regeneration by microfluidic generated fibroblast cell/CuO nanosheet-loaded alginate hydrogel on an excisional full-thickness rat model. ACS Appl Bio Mater 2025;8:3389–403. 10.1021/acsabm.5c00132 40186569

[140] Maldonado VV , Patel NH, Smith EE, Barnes CL, Gustafson MP, Rao RR, Samsonraj RM. Clinical utility of mesenchymal stem/stromal cells in regenerative medicine and cellular therapy. J Biol Eng 2023;17:44. 37434264 10.1186/s13036-023-00361-9 PMC10334654

[141] Nasadiuk K , Kolanowski T, Kowalewski C, Wozniak K, Oldak T, Rozwadowska N. Harnessing mesenchymal stromal cells for advanced wound healing: a comprehensive review of mechanisms and applications. Int J Mol Sci 2024;26:199. 39796055 10.3390/ijms26010199 PMC11719717

[142] Daneste H , Mohammadzadeh Boukani L, Ramezani N, Asadi F, Zaidan HK, Sadeghzade A, Ehsannia M, Azarashk A, Gholizadeh N. Combination therapy along with mesenchymal stem cells in wound healing: the state of the art. Adv Med Sci 2023;68:441–9. 37924749 10.1016/j.advms.2023.10.006

[143] Ghasempour A , Dehghan H, Mahmoudi M, Lavi Arab F. Biomimetic scaffolds loaded with mesenchymal stem cells (MSCs) or MSC-derived exosomes for enhanced wound healing. Stem Cell Res Ther 2024;15:406. 39522032 10.1186/s13287-024-04012-8 PMC11549779

[144] Fitriani N , Wilar G, Narsa AC, Elamin KM, Wathoni N. Alginate-based hydrogels with amniotic membrane stem cells for wound dressing application. Stem Cells Cloning 2025;18:1–13. 39816853 10.2147/SCCAA.S493125 PMC11730520

[145] Huang J , Deng Q, Tsang LL, Chang G, Guo J, Ruan YC, Wang CC, Li G, Chan HF, Zhang X, Jiang X. Mesenchymal stem cells from perinatal tissues promote diabetic wound healing via PI3K/AKT activation. Stem Cell Res Ther 2025;16:59. 39923118 10.1186/s13287-025-04141-8 PMC11807333

[146] Lin L , Liang X, Xu Z, Li Y, Guo Z, Liu L, Liu H, Cai Q, Chen Y, Yu Z, Li Y. Multifunctional hydrogel delivery of mesenchymal stem cell secretome suppresses neutrophil extracellular trap formation and promotes diabetic wound healing via PGE2/BMAL1 pathway. Biomaterials 2026;327:123764. 41092646 10.1016/j.biomaterials.2025.123764

[147] García-Bernal D , García-Arranz M, Yáñez RM, Hervás-Salcedo R, Cortés A, Fernández-García M, Hernando-Rodríguez M, Quintana-Bustamante Ó, Bueren JA, García-Olmo D, Moraleda JM, Segovia JC, Zapata AG. The current status of mesenchymal stromal cells: controversies, unresolved issues and some promising solutions to improve their therapeutic efficacy. Front Cell Dev Biol 2021;9:650664. 33796536 10.3389/fcell.2021.650664 PMC8007911

[148] Di Bella MA. Overview and update on extracellular vesicles: considerations on exosomes and their application in modern medicine. Biology (Basel) 2022;11:804. 35741325 10.3390/biology11060804 PMC9220244

[149] Bian D , Wu Y, Song G, Azizi R, Zamani A. The application of mesenchymal stromal cells (MSCs) and their derivative exosome in skin wound healing: a comprehensive review. Stem Cell Res Ther 2022;13:24. 35073970 10.1186/s13287-021-02697-9 PMC8785459

[150] Rezaei S , Nilforoushzadeh MA, Amirkhani MA, Moghadasali R, Taghiabadi E, Nasrabadi D. Preclinical and clinical studies on the use of extracellular vesicles derived from mesenchymal stem cells in the treatment of chronic wounds. Mol Pharm 2024;21:2637–58. 38728585 10.1021/acs.molpharmaceut.3c01121

[151] Vakilian S , Jamshidi-Adegani F, Al-Fahdi F, Al-Kindi J, Al-Harrasi A, Al-Hashmi S. Optimizing extracellular vesicle delivery using a core-sheath 3D-bioprinted scaffold for chronic wound management. J Vis Exp 2025;216. 10.3791/67764 40095956

[152] Long X , Yuan Q, Tian R, Zhang W, Liu L, Yang M, Yuan X, Deng Z, Li Q, Sun R, Kang Y, Peng Y, Kuang X, Zeng L, Yuan Z. Efficient healing of diabetic wounds by MSC-EV-7A composite hydrogel via suppression of inflammation and enhancement of angiogenesis. Biomater Sci 2024;12:1750–60. 38375548 10.1039/d3bm01904g

[153] Ashrafi F , Emami A, Sefidbakht S, Aghayan H, Soleimani F, Omidfar K. Accelerated healing of full-thickness skin wounds by multifunctional exosome-loaded scaffolds of alginate hydrogel/PCL nanofibers with hemostatic efficacy. Int J Biol Macromol 2025;307:142271. 40112978 10.1016/j.ijbiomac.2025.142271

[154] Rosadas M , Silva IV, Costa JB, Ribeiro VP, Oliveira AL. Decellularized dermal matrices: unleashing the potential in tissue engineering and regenerative medicine. Front Mater 2024;10:2023.

[155] Khosrowpour Z , Bashiri Z, Jafari D, Alizadeh S, Keshtkaran Z, Rezaei B, Bargrizaneh F, Abdollahpour-Alitappeh M, Gholipourmalekabadi M. Translation prospects of a novel ECM-silk fibroin/alginate 3D-Printed scaffold for treatment of full-thickness skin wounds: an in vitro and in vivo study. Polym Adv Technol. 2024;35:e6637.

[156] Karmakar R , Dixit M, Eswar K, Bhattacharjee B, Apoorva B, Gubige M, Sengottaiyan A, Pati F, Rengan AK. Enhanced wound healing properties by sodium alginate-carboxymethyl cellulose hydrogel enriched with decellularized amniotic membrane. Eur J Pharm Biopharm 2025;207:114621. 39725277 10.1016/j.ejpb.2024.114621

[157] Liu Z , Sun W, Su Z, Cui L, Hong Q, Wu D, Li J, Yan Y, Liang H, Tang S, Chen Y, Yu B. Fabrication of a decellularized periosteum matrix-based alginate hydrogel containing collagen for enhanced diabetic wound healing via immuno-neurovascular modulation. Int J Biol Macromol 2025;319:145449. 40545083 10.1016/j.ijbiomac.2025.145449

[158] Su Z , Chen Y, Liang H, Liu Z, Wu D, Li J, Su H, Yan Y, Huang Z, Yu B. Decellularized periosteum and sodium alginate-based photoresponsive dressing with copper selenide nanoparticles for infected-wound healing in diabetic mice. Int J Biol Macromol 2024;268:131895. 38677700 10.1016/j.ijbiomac.2024.131895

[159] Eriksson E , Liu PY, Schultz GS, Martins-Green MM, Tanaka R, Weir D, Gould LJ, Armstrong DG, Gibbons GW, Wolcott R, Olutoye OO, Kirsner RS, Gurtner GC. Chronic wounds: treatment consensus. Wound Repair Regen 2022;30:156–71. 35130362 10.1111/wrr.12994 PMC9305950

[160] Woo KY , Beeckman D, Chakravarthy D. Management of moisture-associated skin damage: a scoping review. Adv Skin Wound Care 2017;30:494–501. 29049257 10.1097/01.ASW.0000525627.54569.da PMC5657465

[161] Yue X , Liu S, Lu B, Zhang X, Dong A, Zhang Y. Research advances in alginate fiber-based medical dressings: a review. Eur Polym J 2026;245:114539.

[162] Varaprasad K , Jayaramudu T, Kanikireddy V, Toro C, Sadiku ER. Alginate-based composite materials for wound dressing application: a mini review. Carbohydr Polym 2020;236:116025. 32172843 10.1016/j.carbpol.2020.116025

[163] Zhang M , Zhao X. Alginate hydrogel dressings for advanced wound management. Int J Biol Macromol 2020;162:1414–28. 32777428 10.1016/j.ijbiomac.2020.07.311

[164] Alhussaini MS , Alyahya AAI, Al-Ghanayem AA. Alginate-derived antibacterial and antifungal agents: a review of applications and advancements (2019-2025). Int J Biol Macromol 2025;318:145333. 40553853 10.1016/j.ijbiomac.2025.145333

[165] Al-Roujayee AS , Hilaj E, Deepak A, Jyothi SR, Hamid JA, Ariffin IA, Saraswat V, Garg A. Alginate-based systems: advancements in drug delivery and wound healing. Int J Polym Mater Polym Biomater 2025;74:846–74.

[166] Fadilah NIM , Maarof M, Motta A, Tabata Y, Fauzi MB. The discovery and development of natural-based biomaterials with demonstrated wound healing properties: a reliable approach in clinical trials. Biomedicines 2022;10:2226. 36140332 10.3390/biomedicines10092226 PMC9496351

[167] Ojeh N , Vecin NM, Pastar I, Volk SW, Wilgus T, Griffiths S, Ramey-Ward AN, Driver VR, DiPietro LA, Gould LJ, Tomic-Canic M. The wound reporting in animal and human preclinical studies (WRAHPS) guidelines. Wound Repair Regen 2025;33:e13232. 39639458 10.1111/wrr.13232 PMC11621255

[168] Vinchurkar K , Bukke SPN, Jain P, Bhadoria J, Likhariya M, Mane S, Suryawanshi M, Veerabhadrappa KV, Eftekhari Z, Onohuean H. Advances in sustainable biomaterials: characterizations, and applications in medicine. Discov Polym 2025;2.

[169] Frykberg RG , Banks J. Challenges in the treatment of chronic wounds. Adv Wound Care (New Rochelle) 2015;4:560–82. 26339534 10.1089/wound.2015.0635 PMC4528992

